# Unveiling the physical mechanism behind pistol shrimp cavitation

**DOI:** 10.1038/s41598-017-14312-0

**Published:** 2017-10-25

**Authors:** Phoevos Koukouvinis, Christoph Bruecker, Manolis Gavaises

**Affiliations:** School of Mathematics, Computer Science & Engineering, City University London, London, UK

## Abstract

Snapping shrimps use a special shaped claw to generate a cavitating high speed water jet. Cavitation formed in this way, may be used for hunting/stunning prey and communication. The present work is a novel computational effort to provide insight on the mechanisms of cavitation formation during the claw closure. The geometry of the claw used here is a simplified claw model, based on prior experimental work. Techniques, such as Immersed Boundary and Homogenous Equilibrium Model (HEM), are employed to describe the claw motion and cavitating flow field respectively. The simulation methodology has been validated against prior experimental work and is applied here for claw closure at realistic conditions. Simulations show that during claw closure, a high velocity jet forms, inducing vortex roll-up around it. If the closure speed is high enough, the intensity of the swirling motion is enough to produce strong depressurization in the vortex core, leading to the formation of a cavitation ring. The cavitation ring moves along the jet axis and, soon after its formation, collapses and rebounds, producing high pressure pulses.

## Introduction

Cavitation in water/liquids is a very effective way of generating shock waves^1^, due to the rapid accelerations/decelerations of the bubble interface during its collapse stage. Cavitation-related phenomena may even appear in nature, in animal species; for example dolphins cannot swim faster than 15 m/s due to cavitation formation^2^, which causes pain. On the other hand, the lack of pain receptors on the fins of fish belonging to the scombrid family^2^ (e.g. mackerels, tunas, etc.) allows them to exceed the cavitation free-limit and cavitation-induced damage has been observed on their bodies. Apart from the hindrance that cavitation may cause to swimming fish, other animal species have evolved to exploit the generation of shock waves through cavitation to stun or kill prey. Examples of such animals are snapping shrimps (belonging to the family of Alpheidae) and mantis shrimps (belonging to the family of Odontodactylidae).

Mantis shrimps have two hammer-like or club-like raptorial appendages, which they use to strike with extreme force their prey, such as e.g. small crustaceans or molluscs. High speed imaging revealed that cavitation may form between the hammer-like appendage and the target^3,4^. It is speculated that the mechanism of cavitation formation is due to the strong depressurization of water due to the Bernoulli principle^3^, i.e. as the fluid moves at high speed, its static pressure drops. Moreover, it is likely that cavitation is enhanced by vortex formation and the hammer rebound after the impact on the target surface^3^. However, there are indications that cavitation in the case of the mantis shrimp may be an unwanted effect. Detailed inspection revealed that cavitation does not only damage the target, but the mantis shrimp’s appendages as well^4^. Over time, the appendage surface becomes pitted and damaged, though frequent moulting of the mantis shrimp replaces the damaged smashing surface. The aforementioned discussion indicates that perhaps in the case of mantis shrimp, cavitation appears to be a side-effect of the percussion, with negative aspects that the shrimp has evolved to handle. On the other hand, it seems that the pistol shrimp is the sole species evolved to actively use cavitation itself as a weapon to kill/stun its prey. The mechanism of cavitation formation in pistol shrimp claws will be analyzed in the present work, focusing on the fluid mechanics aspects of its operation.

**Figure 1:**
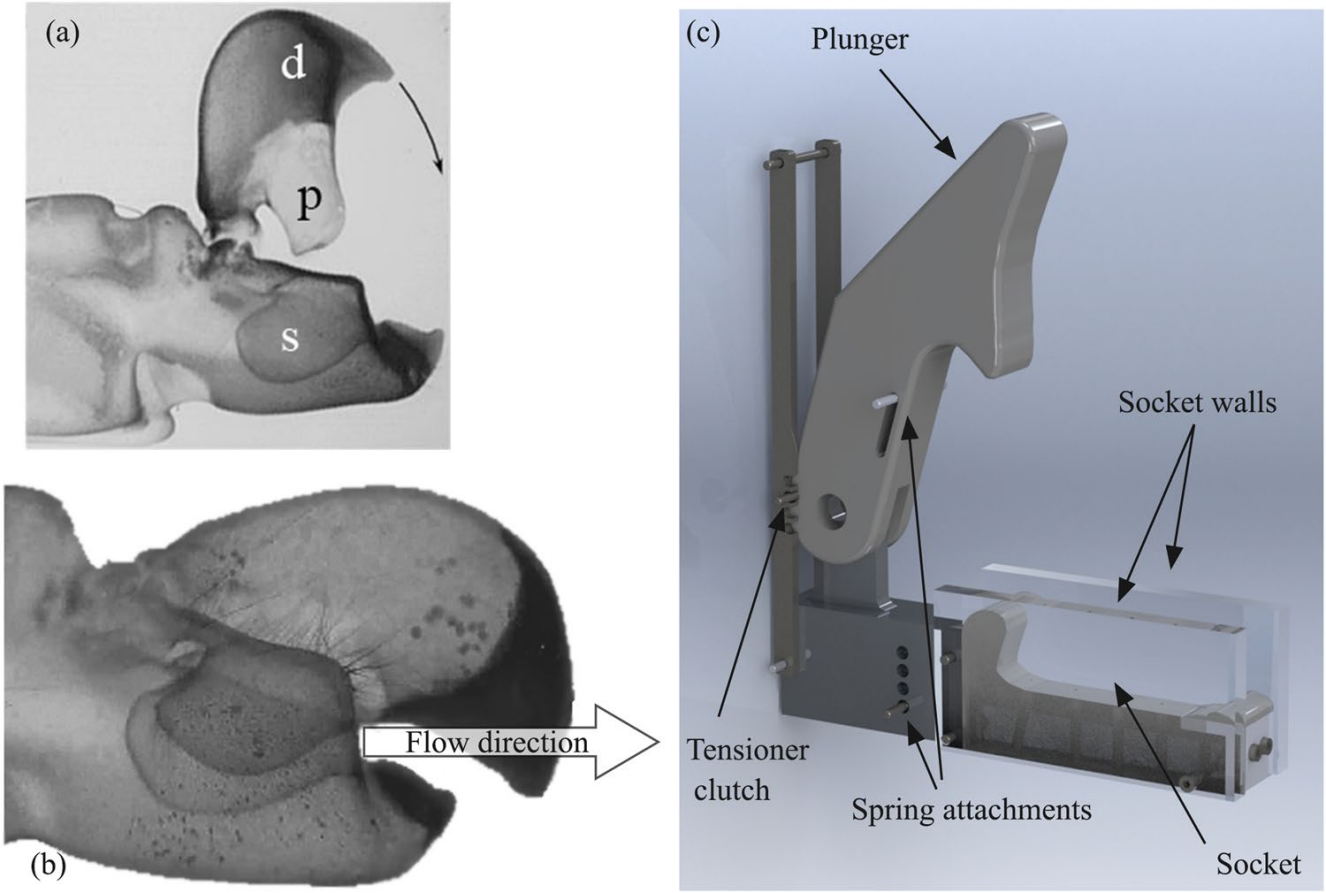
(**a**) Snapping shrimp claw components: *d* corresponds to dactyl, *p* to plunger and *s* to socket^5^. (**b**) Closed claw; the passage through which flow is expelled is visible^5^. (**c**) Render of the simplified claw geometry used in the present study and in previous experimental investigations^5^.

Snapping shrimps, known also as pistol shrimps, have two specially shaped claws, one of which is enlarged and is capable of forming cavitation bubbles^5,6^. Claws are expendable; if the large claw is amputated, the smaller claw will grow to replace the missing limb, whereas a new minor claw will grow in the place of the large claw^7^. The claw consists of two parts, the dactyl and the propus^5^. On the dactyl there is a protrusion (it will be referred as plunger hereafter) which fits into a complementary socket of the propus, see also Fig. 1. When the claw is fully open, water fills the socket of the propus. Then, when the claw closes rapidly, the plunger displaces water from the socket volume. Water escapes through a narrow anterior groove formed between the plunger and the propus, as shown in Fig. 1. The water expelled from the socket through the groove, creates a vortex ring^5^ in a similar way as an air vortex cannon^8^. Note that the shrimp claw is a complicated 3D shape and the expelled jet is not aligned at the same plane as the rest of the claw, thus it is not obstructed by the dactyl tip^9^. Hess *et al*.^5^ introduced the concept of formation number to explain the maximization of momentum transfer from the jet to the vortex. The jet velocity has been estimated by Versluis *et al*.^10^ to be ~25 m/s, using high speed imaging of an actual pistol shrimp claw closing. Such a velocity may lead to pressure drops of ~3^.^10^5^ Pa, which is enough to vaporise water locally^10^ forming a cavitation bubble. Additionally, a simplified numerical investigation, based on the assumption of spherical cavitation bubble solved with the Rayleigh-Plesset equation, indicated pressure levels during collapse of even 2000 bar^10^. Furthermore, a study by Lohse *et al*.^6^ suggests that luminescence phenomena may be observed at the collapsing bubbles formed by pistol shrimps.

While the aforementioned list of experimental work^5,6,10^ aimed to investigate the phenomena being involved in the operation of the pistol shrimp claw, still the mechanism of cavitation formation is not described and well understood. In particular, the work of Versluis *et al*.^10^ examined the macroscopic cavitation formation from the claw and employed a simplified numerical model based on the assumption of spherical bubble shape and relying on parameter fitting to explain cavitation formation. In their work they recognised the lack of detailed flow field and pressure data in the vicinity of the closing claw. The work of Lohse *et al*.^6^ discussed the light emission from collapsing bubbles generated by pistol shrimps, hinting the extreme pressure/temperature conditions during collapse. Not much explanation was provided on the cavitation mechanism or flow field though. Finally, the work of Hess *et al*.^5^ was an experimental study aiming to describe the flow pattern during claw closure by analyzing an enlarged dimensions claw, which was based on a real pistol shrimp claw, scanned using X-ray Computational Tomography (CT). While vortex formation was demonstrated, the enlarged dimensions of the claw geometry did not permit observations of cavitation.

The present work focuses on the fluid mechanics aspects of cavitation formation, growth and collapse, by resolving the flow field around the claw using numerical simulations. The flow field is something that was not analyzed in previous studies, due to experimental limitations. In particular, investigations involving actual pistol shrimps, have constraints in shrimp handling, in the experiment environment and conditions, thus inherently limiting the applicable measurement techniques. High speed photography becomes problematic, since high frame rates are required (of the order of 10^6^ fps), lighting and focusing becomes difficult (the animal may move in a not very controllable manner). The pressure signal recorded from the hydrophone may be excessively smoothed or underestimated by the sensor bandwidth^10^. Moreover, the complexity of the geometry of the claw and the uniqueness of each individual animal, hinder systematic and repeatable study. On the other hand, experimental replicas of pistol shrimp claws lack in reproducing the conditions of cavitation formation; for cavitation to occur, one needs a high speed moving object (the plunger). It is difficult to construct such a plunger in real size dimensions, moving at real closure speed, plus there are difficulties in the experimental techniques (similar to those mentioned above, i.e. high speed imaging, focusing/lighting etc.). This is the reason why Hess *et al*.^5^ resorted to enlarged and non-cavitating conditions.

A general remark in both cases is that experimental techniques such as high speed photography, or pressure signal measurements provide only partial views of the flow pattern and underlying mechanisms. High speed photography can show the existence of cavitation only, but not the actual density of the fluid. Hydrophones may provide information of the pressure signal at a given point, but not everywhere. Particle-Image-Velocimetry (PIV) cannot provide insight in cavitating regions, since the cavitation cloud obstructs the view. The advantage of a well-defined and converged simulation is that it provides a well controlled environment for conducting studies, without limitations of measuring techniques, since they are not necessary (no need for high-speed imaging, Particle-Image-Velocimetry), the flow field is directly accessible in a quantitative manner everywhere. Also there are much less limitations in the simulated conditions and geometry, ensuring repeatability and control. With the above, it is not implied that simulation is the only viable method in conducting research; it is clear that simulation may have pitfalls (hence the clarification “well-defined and converged”). It is also clear that developing simulation tools requires experimentation and theoretical developments to formulate modelling techniques and validate numerical results.

The present work in an attempt to demonstrate the fundamental flow effects occurring at the claw of a pistol shrimp, the mechanism of cavitation generation, shape and collapse. The claw geometry used is based on the simplified model of Hess *et al*.^5^. The reason for resorting to a simplified model is mainly related to validation. There are experimental data available^5^ that can be used to test the numerical methodology (see also supplementary material 3 and 4) and validate the predictive capability of the model before further investigating cavitating conditions. Additionally, the simplified geometry offers the possibility of repeatability in any further research; the geometry is provided as supplementary material (see also supplementary material 12) in Parasolid Computer-Aided Design (CAD) format that can be used by experimentalists to construct their own models, or researchers to develop and test numerical techniques. Note that the methodology employed is applicable for any arbitrary shape, should it be available in a clean Computer-Aided Design (CAD) format.

It is highlighted that in the frame of this work, instead of relying on modelled parameters/fitting, as was the case in the work of Versluis *et al*.^10^, the whole claw and the surrounding fluid are simulated with Computational Fluid Dynamics (CFD). Thus, the present work is the first to simulate the actual flow field inside and outside the claw, demonstrating the flow physics, the cavitation structure and providing additional insight in relation to experiments, since the inherent limitations of the latter are avoided. Despite the simplifications in the claw geometry, the main mechanisms of cavitation generation and collapse are replicable and similar magnitude of jet velocity is found as in experiments involving real pistol shrimps. Briefly stated here, the claw closure produces a high speed jet. The high speed jet induces vortex roll-up, which in turn leads to a strong pressure drop inside the core of the vortex. If the jet velocity is high enough, a pressure drop of even ~10^5^ Pa can be produced, which is enough to vaporize water locally, forming a toroidal cavitation ring. The toroidal cavitation ring oscillates, expanding and collapsing; at the instance of the ring collapse, very high pressures are produced, due to the sudden deceleration of the surrounding liquid.

The simulation of vortex cavitating flows is rather challenging, since high resolution and low numerical dissipation are required to accurately track the vortex^11^. Additionally, cavitating flows are rather difficult to describe and model, due to large pressure and density ratios; in the present simulations, density varies from 998.2 kg/m^3^ (pure liquid) to 0.017 kg/m^3^ (pure vapour) and pressure varies from ~2000 Pa (liquid/vapour mixture) up to 100^.^10^5^ Pa (pressure peaks). These variations have serious implications in the nature of the flow. Strong density variations imply prevalence of compressibility effects, such as low speed shock waves in the bubbly mixture^12^ and pressure pulses in areas of cavitation collapse. Indeed, cavitating flows are known to have a vast variation in the speed of sound, ranging from 0.01 m/s for liquid/vapour mixture up to 10^3^ m/s for pure liquid^13,14^. Cavitation-related computational techniques involve fully Eulerian compressible techniques (selectively^15–17^) or Eulerian-Lagrangian methods (selectively^18–20^). Research on cavitation has many practical applications, ranging from fuel injection systems^21,22^, ship propellers^23^ and pumps^24,25^ to even drug delivery^26^ and cancer treatment^27^. The present research could further promote new and efficient designs in water cleaning/purification devices^28,29^, material processing and chemical engineering^30^.

## Results

Several cases have been examined, for different plunger closure speeds and different plunger sizes. Here, the focus will be on the results of a case with strong cavitation formation to demonstrate the underlying physical mechanisms. The interested reader is addressed to the supplementary material for a complete reference on all cases. The configuration to be presented features a socket with a characteristic length scale of ~1.4 mm and a plunger closure speed of 0.3 ms, resulting to a peak plunger angular velocity of ~7000 rad/s. The Reynolds number of the jet diameter is *Re*_*D*_ ~ 4000 or, based on the plunger length scale, *Re*_*L*_ ~ 40000.

**Figure 2:**
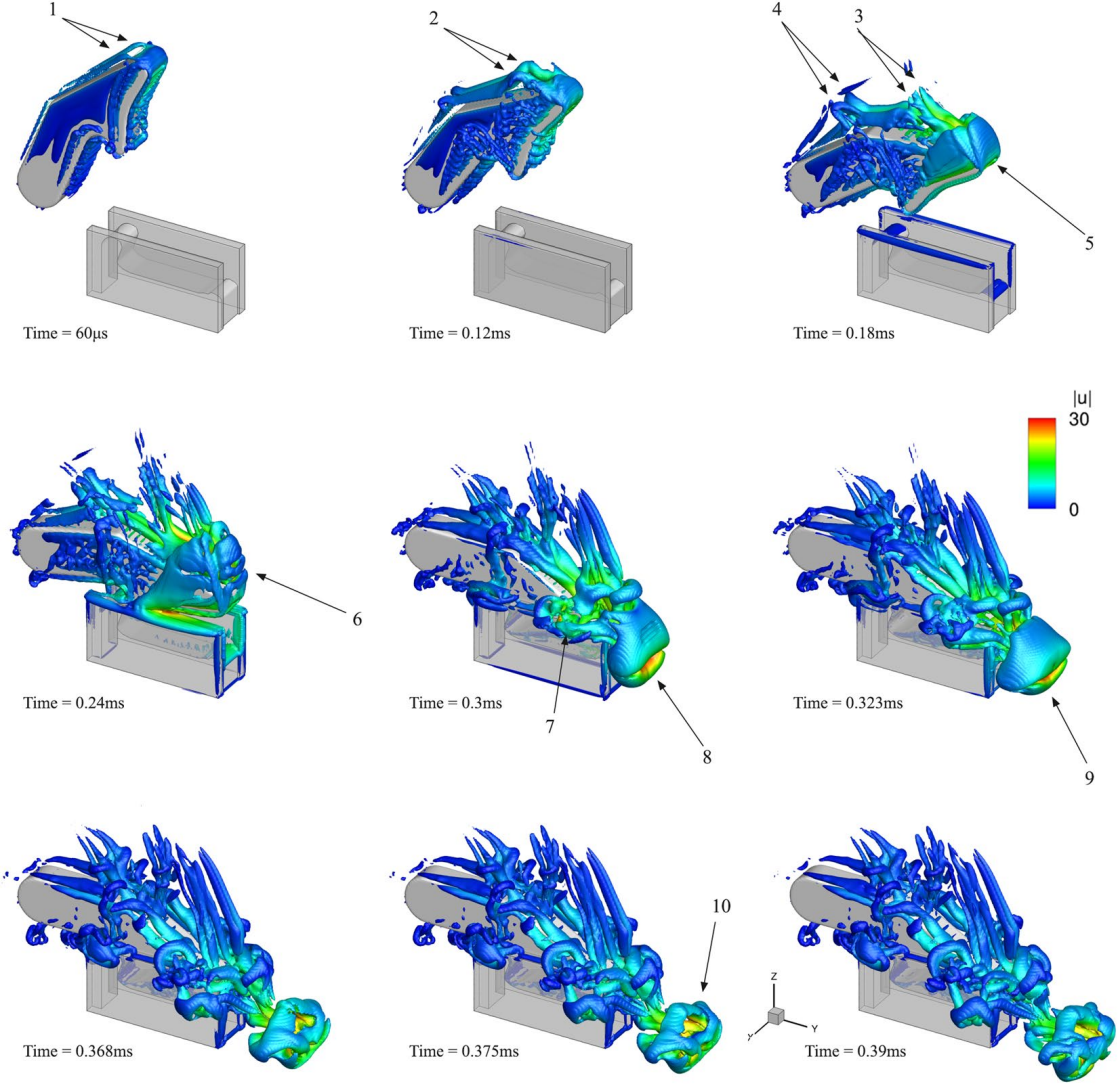
Indicative instances of the ‘real size’ claw model closure; closure time 0.3 ms. Vortices are shown, represented with the velocity gradient second invariant (value *q* = 10^8^ s^−2^), coloured according to the velocity magnitude.

**Figure 3:**
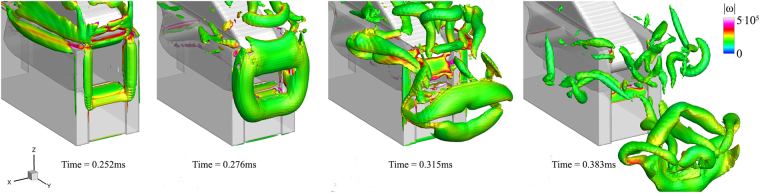
Indicative instances of the vortex ring formation, vortical structures indicated using a q-criterion value of 5^.^10^9^ s^−2^. The isosurface is coloured according to local vorticity magnitude, providing an indication of the swirling angular velocity. Note that due to the square opening between plunger and socket, the vortex ring has initially a square shape as well.

**Figure 4:**
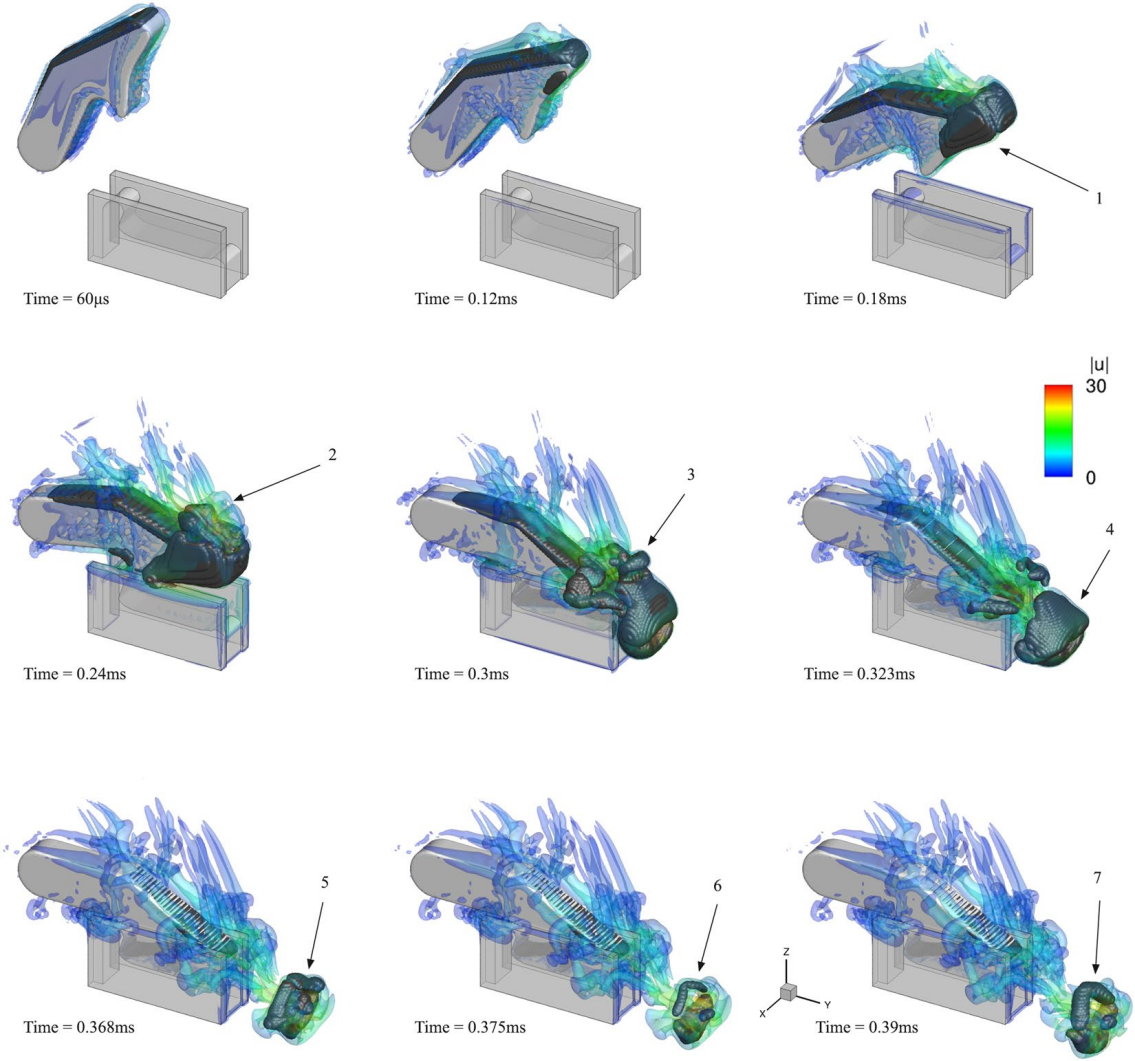
Indicative instances of the simplified claw model closure; closure time 0.3 ms. Vortices are shown, represented with the velocity gradient second invariant (value *q* = 10^8^ s^−2^), coloured according to the velocity magnitude (semi-translucent isosurface). Cavitation is shown with a density isosurface for a value of 990 kg/m^3^ (i.e. vapour vol. fraction ~1% - black opaque isosurface).

The developing vortices during the plunger closure are shown in Fig. 2 and a close-up view around the jet in Fig. 3. Vortical structures are indicated with the isosurface of the *q*-criterion (defined as the second invariant of the velocity gradient tensor^31,32^) for a value of 10^8^ s^−2^. As the plunger starts to move, flow detachment occurs and two counter-rotating vortices form at the wake of the plunger, indicated with (1). As the plunger continues to move, these vortices become larger and start to twist, see (2), (3) and (4). The tip of the plunger is covered by a stretched vortical structure, see (5), occupied by vapour at its core (see also Fig. 4 at the same time instant). Later on, vortex instability^33–36^ leads to break-up of the aforementioned structures, see the wake of the plunger at 0.24 ms or at (6), where the originally stretched vortical structure breaks to several smaller structures. At the same time instant, an attached vortex grows at the wall edge of the socket, due to fluid being expelled from the socket cavity. Because of the closure speed, a high speed jet is expelled from the opening between the plunger and socket walls. The jet velocity is ~30 m/s, inducing vortex roll-up and causing the formation of a large vortex ring, as shown at (8) at 0.3 ms, occupied by vapour due to strong circulation, see (4) at Fig. 4. Vortex roll-up is also observed at the sides of the socket walls, due to liquid escaping from the gap between socket walls and plunger, see (7). After its formation, the vortex ring detaches from the socket/plunger opening and starts to move in the direction of the jet, at a translation velocity approximately half of the jet velocity. The same mechanism is in agreement with experimental observations, see^5,37^. Soon after its formation, the vortex ring elongates, see (9) at 0.323 ms, and then breaks into a complicated vortical structure, see e.g. (10) at 0.375 ms, due to vortex instability, the collapse and rebound of the cavitation ring.

The formation of the vortex ring is shown in detail in Fig. 3. Initially, at 0.252 ms, an attached vortex starts to form at the edges of the geometry, due to the expelled water jet. Note that the rectangular shape of the geometry causes the formation of a rectangular vortex ring as well. Later on, at 0.276 ms, the vortex ring continues to grow and detaches. Its shape still resembles a rectangle, though it is smoothed at corners under the influence of viscosity. At 0.315 ms the vortex ring has completely detached and travels following the jet. Its shape is elongated in the *x*-direction, resembling two cylinders with a gap in between, through which the jet moves. The elongated jet shape is caused by the asymmetric flow field promoted by the plunger motion. Finally, at 0.383 ms, the vortex ring appears shattered after the cavitation ring collapse.

Colouring in Fig. 3 provides an indication of the swirling motion that the fluid is subjected to. The colouring is according to the vorticity magnitude, |*ω*| (defined as the magnitude of the curl of velocity vector field^35^). Under the assumption of forced (or rigid body) vortex type, vorticity and angular velocity are linked. Vorticity is twice the angular velocity of the instantaneous principal axes of the strain-rate tensor of a fluid element^35^. This implies that the liquid is undergoing intense swirling, since angular velocities may range from *Ω* ~ 80000–170000 rad/s. The induced liquid depressurization (defined as pressure at vortex radius *R*, *p*_*R*_, minus the pressure at the vortex core, *p*_*c*_) may be expressed as^13^:

1 $${p}_{R}-p{}_{c}\,=\,\frac{\rho {R}^{2}{{\rm{\Omega }}}^{2}}{2}$$

Considering that the liquid density is *ρ* ~ 998.2 kg/m^3^ and the vortex radius is *R* ~ 0.1–0.3 mm, then the pressure drop ranges between ~5^.^10^4^ up to even 10^6^ Pa, with an average pressure drop of ~2^.^10^5^ Pa. This value is similar to the one used as a fitting parameter by Versluis *et al*.^10^, justifying that despite the simplicity of the model geometry, there is similarity in the underlying physical mechanisms of actual shrimp claws. It should be noted that the forced vortex assumption is not necessarily far from reality, since real fluid vortices are combinations of forced and free vortices. Moreover, this assumption serves to provide an order of magnitude estimate of the angular velocity, explaining the induced liquid depressurization.

In Fig. 4, indicative instances of cavitation formation are shown, combined with the presence of turbulent structures. Turbulent structures are represented as translucent isosurface, whereas cavitation is represented using the density isosurface, for a density value of 990 kg/m^3^ (or vapour volume fraction of ~1%). This combined representation enables to link cavitation structures with vortical structures. At the start of the plunger motion, attached cavitation develops at the wake of the plunger due to local flow detachment. As the plunger accelerates, reaching maximum angular velocity, flow detachment at the sides and the tip of the plunger induces the formation of cavitation sheets, see (1) at 0.18 ms. Later on, detached cavitation structures are observed at the plunger wake at the cores of vortices, e.g. see (2) and (3). The rapid plunger closure leads to the formation of a cavitating vortex ring around the high speed jet, which is clearly shown in (4). After formation, the cavitation vortex ring moves following the jet and oscillates, collapsing and then rebounding again, see the sequence of (5 - collapse), (6 - minimum size) and (7 - rebound). At minimum ring minor radius, at the final stage of collapse and before the cavitation ring rebound, very high pressures are generated, in the order of 100 bar. At the same time, the strong flow acceleration, due to vortex rebound deforms the vortex even more and shatters the cavitation ring.

The generated vortex ring cross-section is a Burgers vortex and its circulation is ~0.005 m^2^/s throughout the whole simulation time. The minor radius of the forced vortex core is ~0.11 mm at generation, later increasing to 0.22 mm after the cavitation ring rebound.

**Figure 5:**
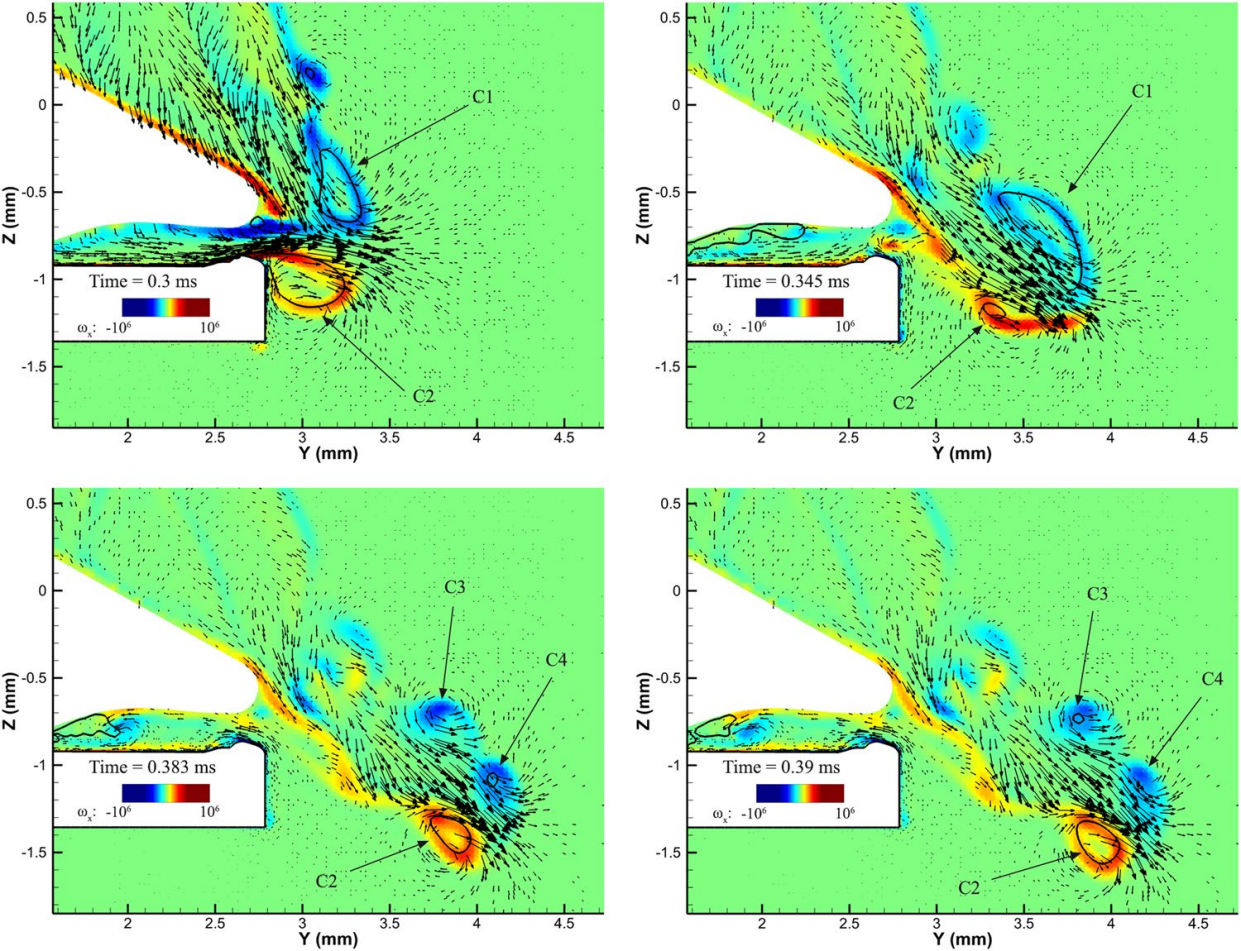
*x*-vorticity (*ω*_*x*_, 1/s) and velocity vectors represented at the midplane (*yz*-plane) of the geometry. The black thick line indicates a density isoline of 500 kg/m^3^ (i.e. vapour volume fraction of 50%).

To demonstrate with clarity the flow field, Fig. 5 shows the flow field at the midplane of the 3D geometry. Flow velocity is represented with velocity vectors whereas the contour shows vorticity at the normal, to the midplane, direction (*ω*_*x*_). Cavitation is represented using a density isoline for a value of 500 kg/m^3^ (or 50% vapour volume fraction). The core of the vortex ring is tracked over time and annotated with arrows.

Instances in Fig. 5 show clearly the correlation of vortex roll-up with cavitation structures; note that at 0.3 ms (plunger closure) cavitation occupies entirely the core of the two counter-rotating vortices, indicating as “C1” and “C2”. At 0.345 ms, “C1” becomes larger, whereas “C2” shrinks, due to the interaction of jet and plunger wake. After the collapse of the cavitation ring, “C1” vortex splits in two. The two new vortices, named “C3” and “C4”, may cavitate alternatively, e.g. at 0.383 ms vortex “C4” cavitates, whereas at 0.39 ms vortex “C3” cavitates.

Plunger motion displaces liquid from the socket, causing the formation of a high speed jet towards the +*y* direction. However, the plunger imparts momentum to liquid at its wake, towards the *−z* direction. Interaction of the jet with fluid from the plunger wake leads to a deviation of jet and cavitation vortex ring from the horizontal direction. Indeed, the jet-wake interaction imparts downward momentum to the jet, which is observable in the presented instances in Fig. 5. Similar effect was observed in the experiment as well and it is demonstrated in the validation study in the supplementary material.

## Discussion

Even though cavitation ring rebounding might seem unexpected, the rebound mechanism is physical and is related to conservation of angular momentum. Indeed, it may be proven that, for a vortex (cylindrical or toroidal), circulation acts in a similar way to a non-linear spring, preventing complete collapse, since the induced centrifugal forces tend to increase the vortex size, eventually leading to rebound, see J.P. Franc^13^. In essence, as long as vorticity is preserved (e.g. inviscid fluid), the cavitation ring would rebound indefinitely. The collapse time for a toroidal cavitation ring may be approximated as^13^:

2 $$\tau \cong {R}_{0}\sqrt{\frac{\rho }{{\rm{\Delta }}p}}\sqrt{\mathrm{ln}\,\frac{8}{\varepsilon }}$$

in the limit of small minor to major torus radius ratio. In equation (2), *R*_0_ is the minor torus radius, *ρ* is the liquid density, Δ*p* is the pressure difference between far field and the cavitating vortex core and *ε* is the ratio between the minor and major torus radii. For the configurations examined in the present work, the R_0_ is ~0.1 mm, Δ*p* ~ 97 kPa, *ρ* ~ 998.2 kg/m^3^ and *ε* ~ 0.16, leading to an oscillation period approximately twice the collapse time, i.e. ~32 μs.

**Figure 6:**
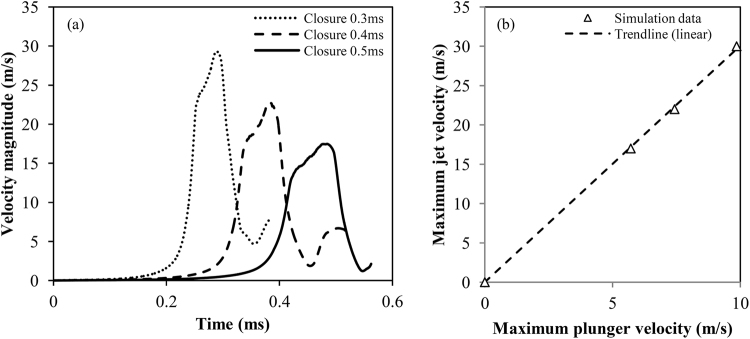
(**a**) Comparison of the flow velocity at the neck, for different closure speeds. (**b**) Relation between the maximum jet velocity and the maximum plunger velocity.

Since in nature pistol shrimps are not identical, it is reasonable to expect variations in the claw size or closure speed. For this reason, a parametric investigation was performed to determine the effect of the closure speed to jet velocity and cavitation volume. In Fig. 6a, a comparison between the jet velocity of several cases is shown, for claw closure times of 0.3 ms, 0.4 ms and 0.5 ms. The angular closure speeds range between 4000 up to 7000 rad/s and plunger velocity at tip between 5.7 up to 10 m/s. Jet velocity is measured at the neck of the formed orifice, as in the experiment^5^. The peak jet velocity is a linear function of the maximum plunger closure velocity (see Fig. 6b). In all cases a local minimum is found after the jet velocity peak, which is closely followed by a second peak, much smaller than the first. This second peak is associated with flow reversal inside the socket. Indeed, during the last stages of the plunger closure, depressurization induced cavitation occurs between the socket/plunger, due to the expelled jet inertia. Thus, shortly after the jet formation, flow rushes back at the cavity formed between the plunger/socket. Indicative instances of the flow reversal are shown in supplementary material.

**Figure 7:**
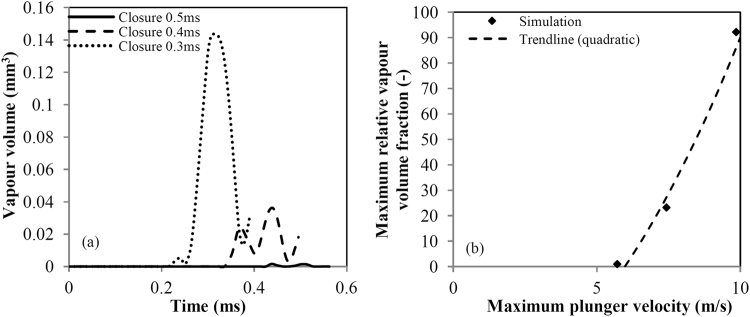
(**a**) Comparison of the vapour volume generated during the plunger motion. Calculation performed as the volume integral of the vapour volume fraction. (**b**) Maximum relative vapour volume defined in respect to the slowest closure speed investigated (i.e. 0.5 ms closure, total vapour volume of 0.00156 mm^3^).

Figure 7 shows the vapour volume in the cavitation ring formed by the plunger closure in respect to time. A global maximum of vapour volume is clearly observed around the time of plunger closure, closely followed by a local minimum due to the cavitation ring rebound. The time scale of the ring rebound is ~70 μs, close to the calculated period from equation (2). Discrepancy is expected, mainly because equation (2) is applicable for small minor to major torus radius ratio and a perfectly circular ring, which is obviously not the case here.

The maximum volume of vapour is related to the closure speed as a quadratic function of the form $V(u)=a{u}^{2}+b$, see Fig. 7b. This form resembles the dynamic pressure contribution (0.5 *ρu* ^2^), including a constant value which is related to the vaporization pressure threshold. As already demonstrated, the plunger speed is linearly related to the jet speed. The jet speed affects the pressure inside the vortex core, since vortex pressure is a quadratic function of tangential vortex velocity^13^. It is highlighted that Fig. 7 discusses only cavitation volume in the ring, omitting cavitation formed at the wake of the plunger or inside the socket, since the latter may not be relevant to the actual shrimp claw, due to differences in the exact claw shape. In any case, for the sake of completeness, it is mentioned that the trend relating maximum vapour volume in the whole computational domain to the closure speed is similar to the one shown in Fig. 7b.

**Figure 8:**
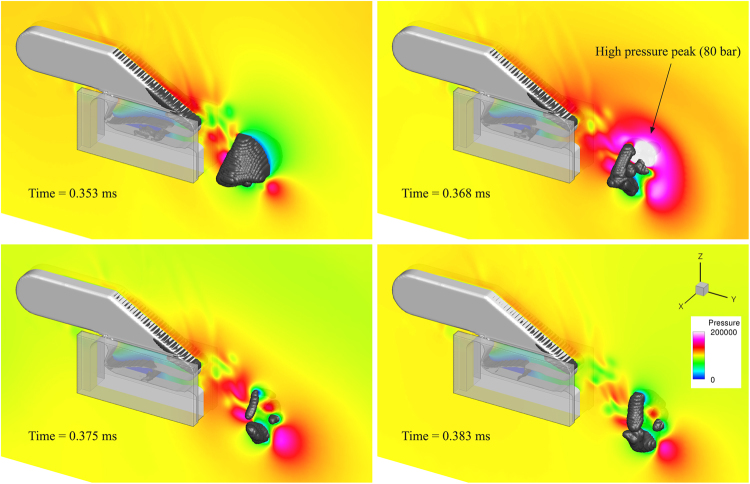
Pressure peak due to cavity collapse, plunger closure at 0.3 ms. Pressure is shown at a midplane slice. The black isosurface is the 1% vapour fraction. Pressure, locally, may exceed 80 bar.

As the cavitation ring collapses and rebounds, very high pressures are produced due to sharp deceleration of surrounding liquid. In essence, the sudden deceleration of liquid results to a water-hammer effect, consequently emitting a pressure pulse. This pressure pulse is the speculated mechanism employed by the pistol shrimp to stun or kill its prey^10^. The generated pressure peak is closely related to the amount of vapour produced during the plunger closure. When the plunger moves at the highest speed examined here (closure at 0.3 ms, max. angular velocity 7000 rad/s, see *Results* section), an intense pressure peak is found, reaching instantaneous pressures of even 80 bar, see Fig. 8.

**Figure 9:**
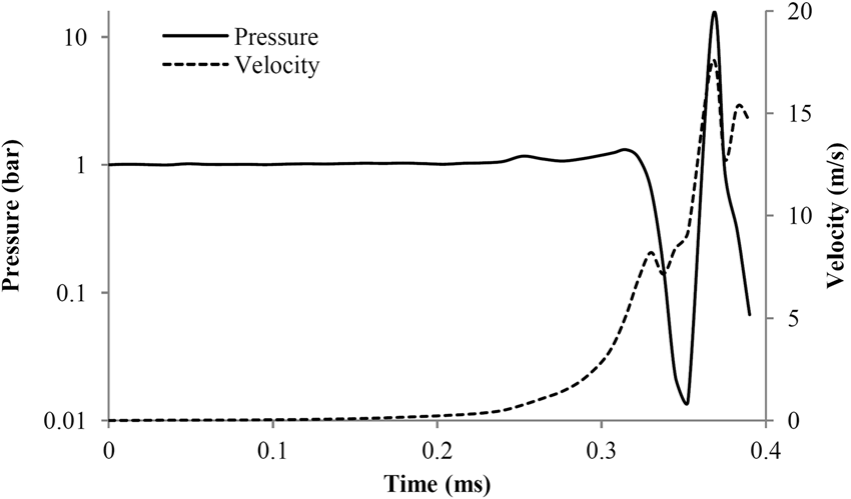
Pressure and velocity magnitude as a function of time, at a characteristic length scale *L* = 1.4 mm from the claw neck.

Figure 9 shows the time evolution of pressure and velocity magnitude at a characteristic length scale *L* ~ 1.4 mm (see Table 1) away from the claw neck, at the *y*-direction, for plunger closure at 0.3 ms. Before the time of 0.2 ms, pressure signal is almost stable. Then, from 0.2 to 0.3 ms small pressure peaks are detected, followed by a sudden pressure drop at 0.35 ms. At the instance of cavitation ring collapse a very high pressure pulse is found, reaching pressures of more than 10 bar. At the same time instant there is a local maximum of flow velocity, reaching 17 m/s. The pressure peak is then followed by a second pressure drop. The pressure signal pattern is the same as the one found in the prior work by Versluis^10^.

**Table 1:** Characteristics of the real size and enlarged models examined^5^.

	Experiment - ‘enlarged model’	‘Real size’
Geometry – *L* (socket length scale)	0.1 m	1.41 mm
Liquid dynamic viscosity - *μ*	5 mPa.s	1 mPa.s
Density - *ρ*	998.2 kg/m^3^	998.2 kg/m^3^
Closure time - *t*_*closure*_	0.5 s	0.5 ms
Indicative Velocity - *u*	~1 m/s	~17 m/s
Reynolds number - *Re*_*L*_	~20000	~20000

To summarize, the present work is the first to analyze the cavitating flow in a geometry resembling a pistol shrimp claw, providing insight in the physical mechanisms of cavitation generation and proving that cavitation produced by the shrimp claw is not a spherical bubble but rather a toroidal cavitation structure. The main mechanism of the cavitating claw operation is vortex ring roll-up, induced by the high speed jet expelled from the socket. Depending on the plunger closure speed, circulation of the vortex ring may become high enough to cause a considerable pressure drop inside the vortex core. A large pressure drop may induce vaporization of the liquid inside the vortex core, leading to the formation of a cavitating vortex ring. Upon its formation, the cavitation ring travels at the direction of the jet, with a translational velocity around half of that of the jet and its minor radius oscillating until viscosity dissipates angular momentum. The oscillation of the cavitation ring leads to periodic collapses and rebounds, which emit high pressure pulses. These pressure pulses are used by the shrimp for communication, as a defence mechanism, to stun, or kill the shrimp’s prey.

Considering all the aforementioned observations, similarities and differences of the flow produced by a simplified and an actual pistol shrimp claw may be summarised. First of all, from the results it is clear that, as the claw plunger moves inside the socket, the displaced liquid forms a high velocity jet, which in turn induces vortex ring roll-up. The shape of the vortex ring will affect the shape of cavitation in the vortex core. While in the simulation the vortex ring is rectangular, due to the square shape of the plunger-socket opening, in reality the shrimp’s claw opening is a smooth curve leading to a more circular vortex ring. In the simulation, cavitation at the wake of the plunger was observed. In reality, the streamlined shape of the claw means that flow detachment is limited, thus there is very little cavitation, if any. Moreover, whereas in simulation the socket was fixed in place, in actual pistol shrimp claws both plunger and socket move at opposite directions, offsetting somewhat the jet deviation introduced by the plunger wake. Despite these differences, quantitative characteristics of claw operation have been reproduced. In particular, the maximum plunger angular closure speed in the simulation was 7000 rad/s, whereas actual claws^10^ close at comparable speeds of 3500 rad/s. Plunger closure results to water jet speed of 28–31 m/s predicted by the simulation, whereas measurements^10^ in real claws indicate jet velocities of 25–32 m/s. The pressure drop predicted by the intense swirling motion of the liquid is very similar to the one imposed as fitting parameter by Versluis *et al*.^10^ (simulation ~2 · 10^5^ Pa, reference 2.2 · 10^5^). Moreover, the peak pressure measured from the bubble collapse is comparable to the one found from the present study, see P. Krehl^1^, and the pressure signature is very similar to that measured by Versluis *et al*.^10^. It is also highlighted here, that effects found in the simulations may be confirmed by early investigations of other researchers, working on similar simplified claw models under cavitating conditions, see the work of Eliasson *et al*.^38,39^. To be more specific, the downwards deflection of the jet and the cavitation ring, the formation of cavitation at the wake of the plunger and the formation of cavitation inside the plunger/socket cavity are clearly shown in high speed videos^38,40^, providing additional validation of the presented results.

## Methods

The numerical methodology used in the present work is discussed in detail in the supplementary material, but will be described here briefly. The plunger motion is imposed using an Immersed Boundary (IB) technique^41–43^. The advantage of this technique is that the computational domain remains unchanged throughout the whole simulation time, thus greatly simplifying geometry manipulation, especially in cases of small gaps or contact regions. Cavitation is modelled using the Homogenous Equilibrium Assumption^15,44–46^, thus pressure and density are directly linked through an Equation of State (EoS) describing the phase change process. This assumption is justified based on cavitation tunnel experiments^47^.

The geometry used for the simulations is based on prior experimental studies^5^. Experiments were based on the claw morphology of a typical specimen of snapping shrimp, A. bellulus. The morphology of the claw was obtained in a computerized form using X-ray micro-Computed Tomography (μ-CT) scanning, at fully closed and open positions. A two dimensional slice was extracted along the midplane of the claw geometry, obtaining the mean profile of plunger and socket geometry. This two dimensional slice was extruded in the 3rd direction, to obtain a simplified model of the shrimp claw. Additionally, scale similarity was exploited to manufacture an enlarged scale model of the claw (scale 70:1), which has been used for experimental studies, involving flow visualization and Particle Image Velocimetry. In the scope of the present study, two types of simulations have been performed. One simulation involved the ‘enlarged model’ geometry that was used in previous experiments, at the same conditions (e.g. plunger closure profile). The aim of this simulation was to validate the numerical framework and detailed results are presented in the supplementary material. The second set of simulations involved parametric studies of the ‘real size’ geometry, based on the dimensions of the actual snapping shrimp claw. Results of the second set of simulations are presented in this paper, since they involve cavitation related effects which are the focus of the study.

**Figure 10:**
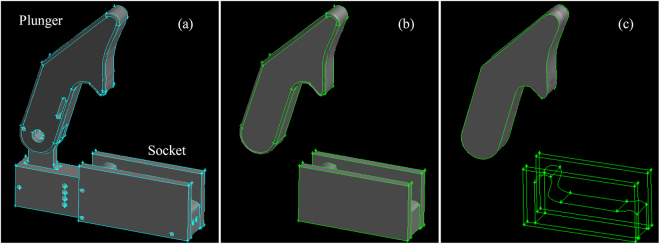
Left to right: (**a**) original geometry, used for enlarged scale experiments, (**b**) simplified geometry (hole and small features removed) and (**c**) final geometry (fillets removed). In (**c**) the wireframe of the socket is shown, providing a view to the inner geometry of the socket.

As shown in Fig. 10a, the experimental geometry has many construction features, such as holes for spring attachments, hinge shaft etc. Such features are not necessary for the simulation, since the area of interest is in the flow channel between plunger and socket. Thus, such features have been removed (Fig. 10b). Moreover, the fillet of the geometry has been removed (Fig. 10c), for simplifying the triangulation of the plunger surface, which is needed for preparing the marker point set (see supplementary material 1). The plunger initially is positioned at 73° from the fully closed configuration.

The simplified pistol shrimp claw dimensions, jet velocity and Reynolds number are outlined in Table 1.

The Reynolds number may be defined based on the socket length scale, *L*, as in the experiment^5^ for consistency:

3 $${\mathrm{Re}}_{L}=\frac{u\cdot L\cdot \rho }{\mu }$$

It is highlighted though, that the velocities reported in Table 1 occur in the neck region of the formed nozzle, as the claw closes. Thus, one could define the Reynolds number, based on the jet diameter, *D*, which is comparable to the nozzle neck, i.e. ~1 cm for the ‘enlarged model’ or ~ 0.14 mm for the ‘real size’ model, as:

4 $${\mathrm{Re}}_{D}=\frac{u\cdot {D}_{jet}\cdot \rho }{\mu }$$

Based on the nozzle dimensions, the jet Reynolds number is *Re*_*D*_ ~ 2000 for both ‘real size’ and ‘enlarged model’ cases. The maximum jet Reynolds number of the parametric cases examined is ~4000, thus the developed flow is laminar or at the borderline to transitional, consequently an explicit turbulence model was not used.

### Data availability

The data used for the present study are included as supplementary materials:

- The claw geometry is included in Supplementary material 12 in Parasolid CAD format.
- The motion profile is presented in Supplementary material 3.

The aforementioned data are adequate to define a simulation or design an experiment. In case additional information are required, the interested reader is addressed to the corresponding author (see below).

## Electronic supplementary material

Supplementary material 8

Supplementary material 8

Supplementary material 11

Supplementary material 11

Supplementary material 10

Supplementary material 10

Supplementary material 9

Supplementary material 9

Supplementary material
