# Supplementary material for: Unveiling the physical mechanism behind pistol shrimp cavitation
Source: Sci Rep. 2017 Oct 25;7:13994. doi: 10.1038/s41598-017-14312-0 (PMC5656667; doi:10.1038/s41598-017-14312-0)
Supplement: Supplementary file 9 — Supplementary material [file 41598_2017_14312_MOESM9_ESM.doc]

Unveiling the physical mechanism behind pistol shrimp cavitation

Phoevos Koukouvinis*, Christoph Bruecker, Manolis Gavaises

School of Mathematics, Computer Science & Engineering, City University London

E-mail: Foivos.Koukouvinis.1@city.ac.uk

**Supplementary material 1: Simulation methodology**

Simulation methodology is based on a Homogenous Equilibrium Mixture (HEM) assumption for modelling cavitation effects. Such a methodology has been successfully employed in the past (e.g. selectively [^1-4^](#_ENREF_1)) for cavitating flows and bubble dynamics. The HEM technique has the advantage of simplifying the system of equations to be solved, since density changes are related directly to pressure, thus a vapour fraction transport equation (as in mass transfer-based models, e.g. ) is redundant. The mixture continuity and momentum equations are solved:

$\frac{\partial\rho}{\partial t}+\nabla\cdot\left(\rho\mathbf{u}\right)=0$ (1) $\frac{\partial\rho\mathbf{u}}{\partial t}+\nabla\cdot\left(\rho\mathbf{u}\otimes\mathbf{u}\right)=-\nabla p+\nabla\cdot\text{τ}+\mathbf{f}_{\mathbf{IB}}$ (2)

where *ρ* is the mixture density, ***u*** is the velocity vector, *p* is pressure and **τ** denotes the stress tensor, as described below:

$\text{τ}=\mu\left[\nabla\mathbf{u}+\left(\nabla\mathbf{u}\right)^{T}\right]+\lambda\left(\nabla\cdot\mathbf{u}\right)\text{ I}$ (3)

with *μ* the viscosity of the mixture and **I** is the identity matrix. The effect of bulk viscosity, *λ*, is omitted from equation 3, due to the small effect it has to the flow dynamics, since it acts only on passing waves [^7^](#_ENREF_7).

Due to the tight gaps between the socket and the claw plunger, which would render simulation with deforming/adaptive meshes very computationally inefficient, the immersed boundary technique was employed for imposing the plunger motion. The immersed boundary technique employed here is based on the continuous forcing method [^8-10^](#_ENREF_8). In practice, a source term is included in the momentum equation, which is the term ***f_IB_*** in equation (2). This term forces the flow to move following the solid body's motion and is formulated as follows:

$\mathbf{f}_{\mathbf{IB}}=-c_{str}\frac{a_{w}}{dt}\left(\mathbf{u}-\mathbf{u}_{t\arg et}\right)\rho$ (4)

The forcing term includes the contribution of the wall volume fraction *a*_w_ since a given cell may be only partially occupied by wall, time step *dt* and is proportional to the difference between the flow velocity and the target body velocity. A tuning factor, *c_str_*, is included to adjust the strength of the immersed body velocity; the larger the strength, the smaller the deviation from the target velocity ***u****_target_*. In the present simulations, the target velocity and immersed body motion were imposed based on the linear interpolation of the angle and angular velocity profile provided in *Supplementary Material 3*, depending on the examined case. All present simulations have been performed with a tuning factor, *c_str_*, value equal to unity.

To identify the wall volume fraction, *a_w_*, at each cell, the distance of cell nodes from the immersed body, at the wall normal direction, needs to be calculated. This is done by looping all cell nodes and identifying which immersed body marker point is at minimum distance; this point is used to calculate the node distance at normal direction, *d_n,IB_*, from the immersed body, as follows (see also Figure 1):

$d_{n,IB}=\left(\mathbf{x}_{\mathbf{n}}-\mathbf{x}_{IB}\right)\cdot\mathbf{n}_{IB}$ if $\min(\left\|\mathbf{x}_{n}-\mathbf{x}_{IB}\right\|)$ (5)

The immersed boundary wall volume fraction, *a_w_*, in each cell is approximated as follows:

$a_{w}=\frac{\sum_{n} \left[-\min\left(d_{n,IB},0\right)\right]}{\sum_{n} \left|d_{n,IB}\right|}$ (6)

The cell distance to the immersed body, *d_c,IB_*, is estimated as the average node distance to the immersed body, for the nodes that belong to the examined cell, i.e.:

$d_{c,IB}=\frac{\sum_{n} d_{n,IB}}{n}$ (7)


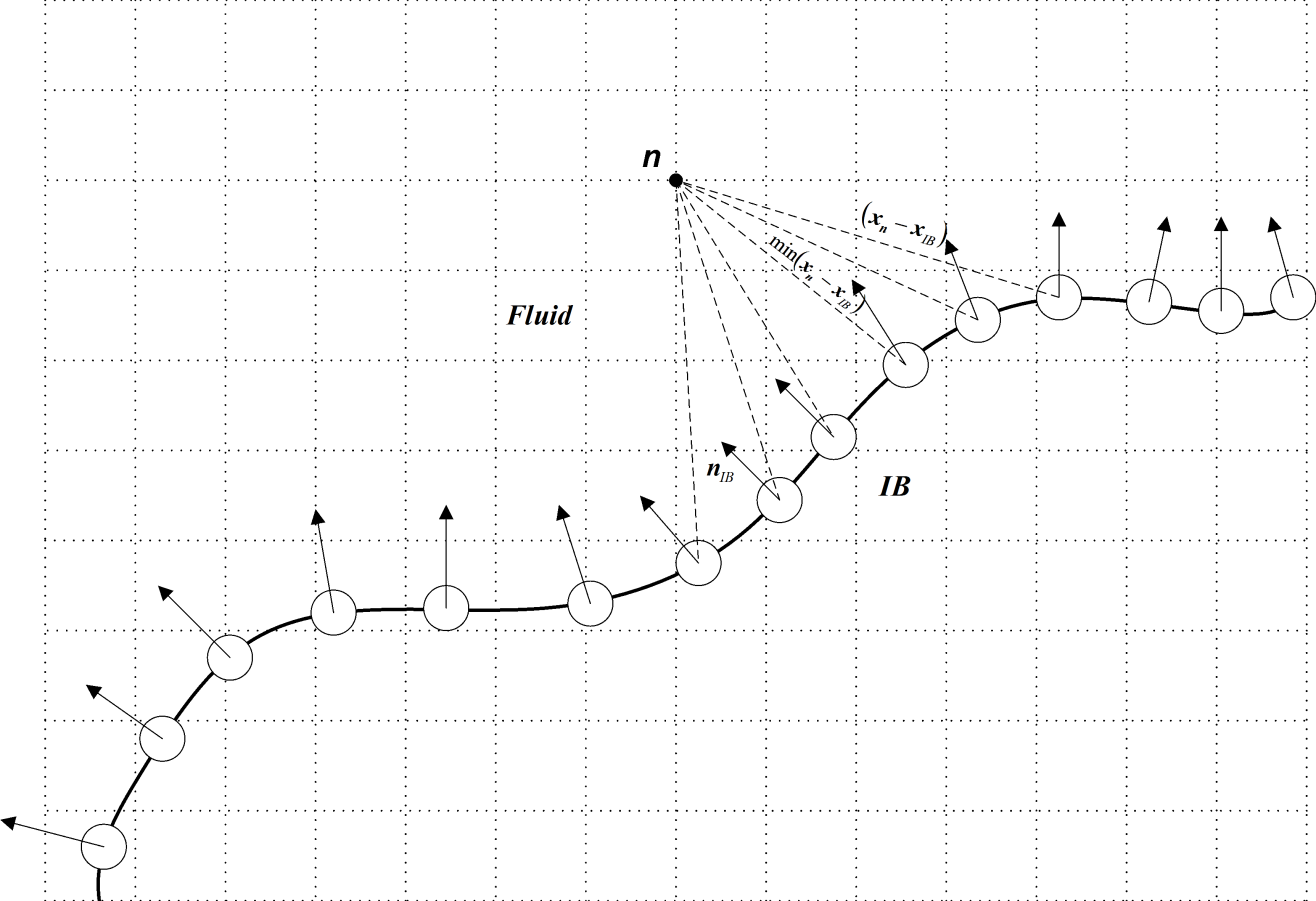

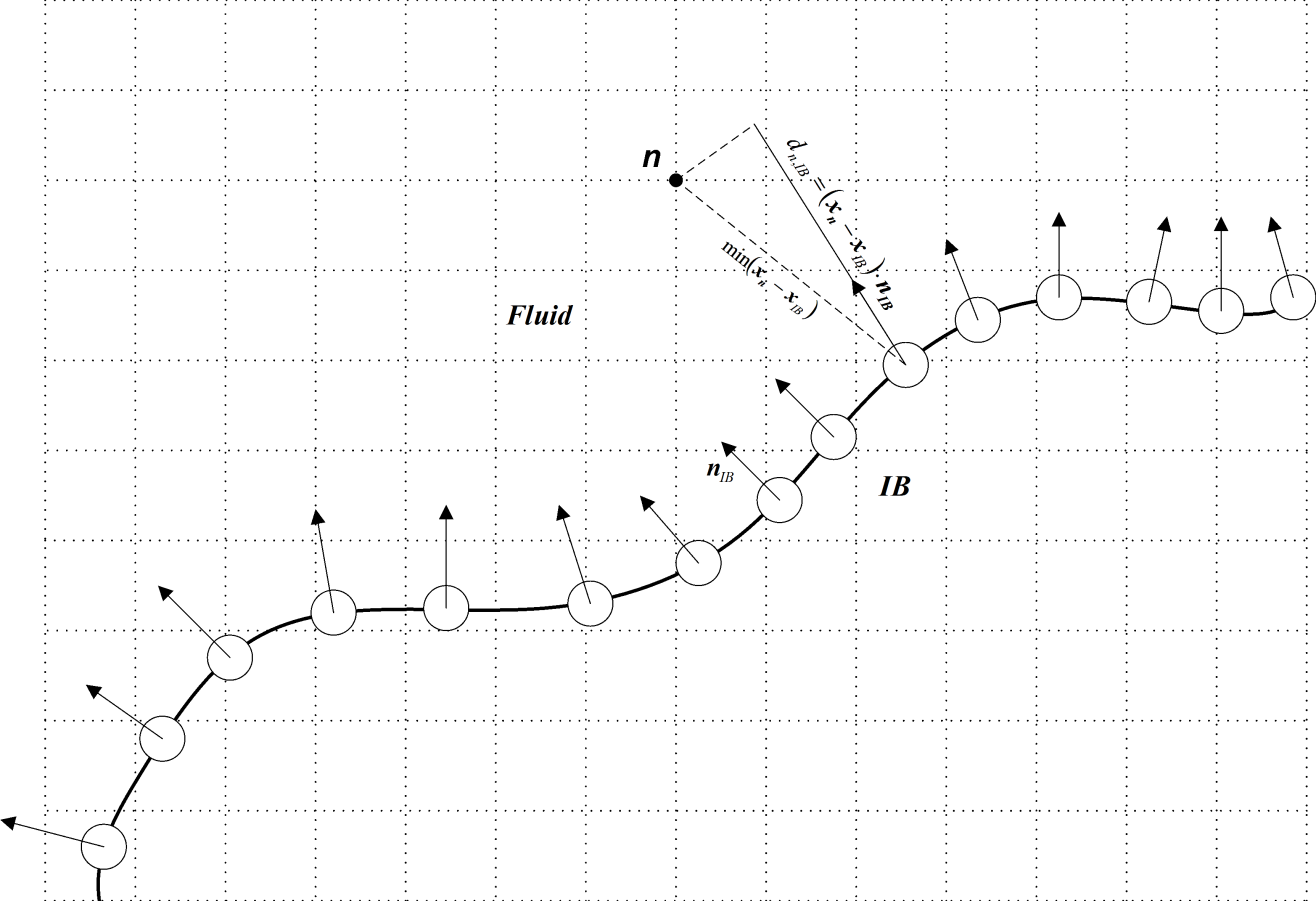


(a)

(b)

Figure 1. Schematic representation of wall distance calculation: (a) closest marker point identification to a given cell node *n*, (b) normal to the wall distance calculation for the given marker point.

The body is converted to a triangulated surface representation, e.g. an STL file. The triangulated surface representation consists of a set of triangular elements, as shown in Figure 2a. The triangulated mesh is converted to a set of marker points, each positioned at the barycentre of the corresponding triangle and associated with the local normal vector, ***n_IB_*** ( Figure 2b). At each time step, the location of the marker points and wall normals, representing the immersed body is updated, based on the motion type (e.g. here rotation). Then the aforementioned procedure, for calculating node and cell distance to immersed body (*d_n,IB_* and *d_c,IB_* respectively) and wall volume fraction, *a_w_*, is performed. Finally, the source term ***f_IB_*** is calculated and integrated to the momentum equation. Despite the simplicity of the described methodology for implementing the immersed boundary contribution, wall velocity was enforced with a deviation from target of less than 1% and the wall volume was conserved with an error of less than 0.01% in all simulations performed so far.


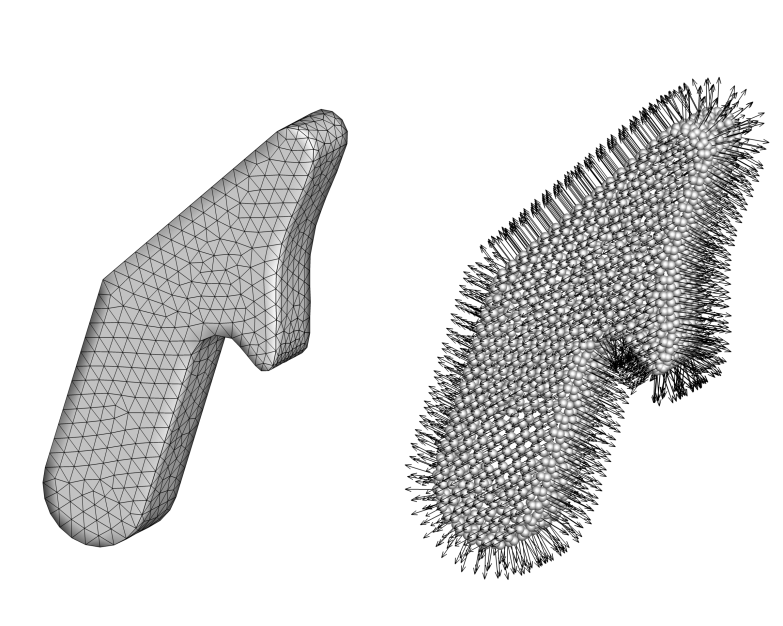


(a)

(b)

Figure 2. (a) Triangulated plunger surface (b) plunger surface represented with a set of marker points (represented as spheres) and wall normal vectors.

As mentioned in the beginning of this section, the HEM assumption is employed. This means that cavitation is described with an appropriate equation of state, capable of predicting the density variation during vaporization. Here the dependence of temperature to pressure will be ignored, thus pressure is linked solely to density; such models are commonly known as barotropic models. Many formulations for such models exists (e.g. [^11-13^](#_ENREF_11)). Here we use the following formulation for the barotropic model [^4^](#_ENREF_4):

$p\left(\rho\right)=\left\{\begin{matrix} B\left[\left(\frac{\rho}{\rho_{l}}\right)^{n}-1\right]+p_{sat,L}\text{ }\rho\geq\rho_{l} \\ \frac{c_{v}^{2}c_{l}^{2}\rho_{l}\rho_{v}\left(\rho_{v}-\rho_{l}\right)}{c_{v}^{2}\rho_{v}^{2}-c_{l}^{2}\rho_{l}^{2}}\ln\left(\frac{\rho}{c_{l}^{2}\rho_{l}\left(\rho_{l}-\rho\right)+c_{v}^{2}\rho_{v}\left(\rho-\rho_{v}\right)}\right)+p_{ref}\text{ }\rho_{l}\geq\rho>\rho_{v} \\ \begin{matrix} \\ C\rho^{\gamma}\text{ }\rho_{v}>\rho\end{matrix} \end{matrix}\right.$ (8)

The vapour volume fraction is defined as:

$a_{v}=\frac{\rho-\rho_{l}}{\rho_{v}-\rho_{l}}$ (9)

and the mixture viscosity:

$\mu=a_{v}\mu_{v}+\left(1-a_{v}\right)\mu_{l}$ (10)

The fluid properties are detailed in table 1. For the properties combination the barotropic equation of state is shown in Figure 3. In equation (8), *p_ref_* and *p_sat,L_* are reference values properly chosen to make sure that pressure is a continuous function of density, thus $\lim_{\rho\to\rho_{v}^{+}} p\left(\rho\right)=\lim_{\rho\to\rho_{v}^{-}} p\left(\rho\right)$ and $\lim_{\rho\to\rho_{l}^{+}} p\left(\rho\right)=\lim_{\rho\to\rho_{l}^{-}} p\left(\rho\right)$. It becomes obvious from the formulation of the equation, that during the phase change there is a small pressure difference equal to Δp=$p_{sat,L}-p_{sat,V}$. In practice, this difference is small in comparison to the pressure levels involved in the simulation, e.g. for the present case, the difference is around 4500 Pa, whereas the pressure level in the current simulation is of the order of ~10^5^ Pa. Moreover, while it is true that the equation of state is not perfectly accurate for the sharp change of pressure in the saturation dome, it has the advantage of having a continuous speed of sound, which helps achieving convergence with the pressure-based solver utilized.

Table 1. Barotropic fluid properties; values have been selected an saturation properties of water/steam at 20^o^C (X-Steam [^14^](#_ENREF_14)) and Ivings et al. [^15^](#_ENREF_15).

| Liquid properties | | | Vapour properties | | |
| --- | --- | --- | --- | --- | --- |
| *B* | 307.1.10^6^ | Pa | *C* | 27234.7 | Pa/(kg/m^3^)^γ^ |
| *n* | 7.15 | (-) | *γ* | 1.33 | (-) |
| *ρ_sat,L_* | 998.16 | kg/m^3^ | *ρ_sat,V_* | 0.0171 | kg/m^3^ |
| *c_sat,L_* | 1483 | m/s | *c_sat,V_* | 97.9 | m/s |
| *p_sat,L_* | 4664.4 | Pa | *p_sat,V_* | 125 | Pa |
| *μ_L_* | 10^-3^ | Pa.s | *μ_V_* | 9.75.10^-6^ | Pa.s |


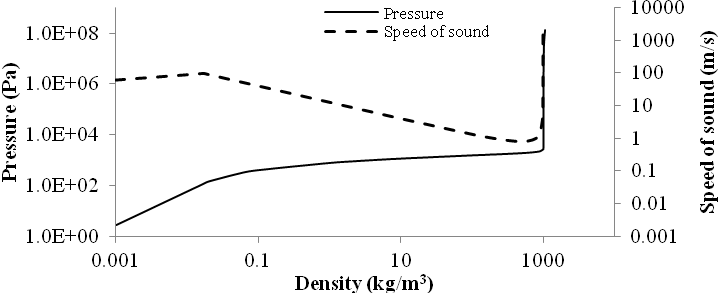


Figure 3. Graph representing the pressure (equation 8) and speed of sound as function of density.

**Supplementary material 2: Discretized geometry and numerical settings**

In this section, the features of the modelled geometry, computational mesh, resolution and numerical settings will be discussed.

All simulations have been carried out with the ANSYS Fluent software [^16^](#_ENREF_16) v. 16.2, modified with User Defined Functions (UDFs) to incorporate the immersed body motion and the barotropic cavitation model. The coupled, pressure-based solver has been used, since it offers good convergence characteristics, despite the problem complexity (see e.g. [^17^](#_ENREF_17)). The spatial discretization used for density and momentum was second order upwind, whereas a body-force weighted scheme was used for pressure interpolation [^18^](#_ENREF_18). Temporal discretization was implicit, first order, with a time step dependent on the maximum expected velocity during closure; the maximum convective courant number was kept below 0.1 in all cases and the acoustic courant was maintained at ~100 for the enlarged, non-cavitating configuration and ~5 for the 'real size' cavitating configuration. Fixed pressure was used for the pressure farfield, and no-slip wall boundary conditions for walls.

The computational mesh for the 3D 'enlarged model' simulations is shown in Figure 4. The mesh is cartesian, cut-cell [^19^](#_ENREF_19), with telescopic refinement around the plunger/socket and near wall refinement at the socket surface. The computational mesh has a base resolution of 14 mm, refined to 1.75mm in the vicinity of the plunger/socket and further refined to 0.7mm near the socket surface. The 3D 'real size' model is identical to the 'enlarged size model', with the only difference that it is scaled by 1:70. Thus, for the 'real size' model the base resolution is 0.2 mm, 0.025 mm around the plunger/socket and 0.01 mm at the vicinity of the socket wall. The total cell count is 7.5M cells.


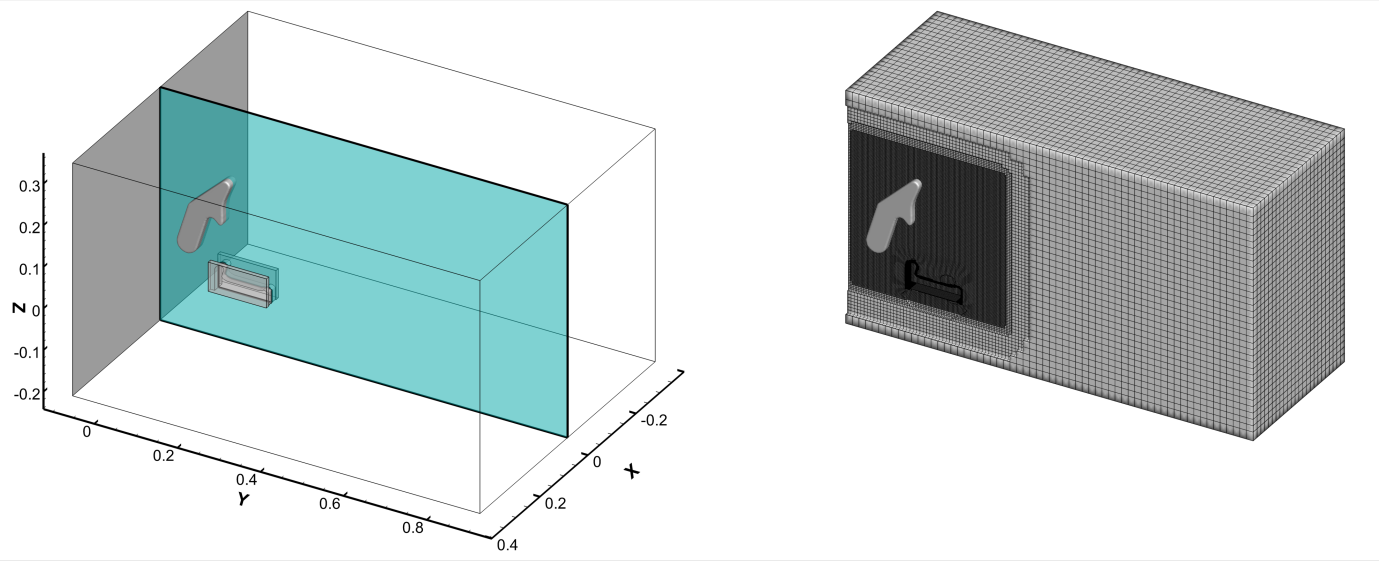


Figure 4. Computational domain and computational mesh for the enlarged 3D case. Grey colour corresponds to wall no-slip boundary conditions, note that the plunger isosurface is also shown grey though it is an immersed body. The rest boundaries are pressure far fields. Telescopic refinement is visible in the vicinity of the plunger/socket; inflation layers are placed near the socket walls.

**Supplementary material 3: Angle data from high-speed videos**

The plunger closure velocity was obtained through high-speed videos, recorded at 300 frames per second and provided by Hess et al. [^20^](#_ENREF_20) as supporting material. The plunger had a white marker, for easier recognition of the angular position (see also Figure 5a at the bottom part of the plunger, along the dashed path). The methodology for deriving the closure angular velocity is the following:

- As a first step, the arc along which the white marker moves is found, based on three indicative frames. As shown in Figure 5a, one frame corresponds to the first instance the marker is within the camera's optical field, another frame at the middle of the plunger path and a final frame at the fully closed position.

- The next step is to calculate the closure angle from the fully closed position for different frames of the high speed video. Since each frame is obtained with a temporal sampling of 1/300s, correlation between the frame number and time is direct.

- The closure angular velocity is found, by differentiating the plunger closure angle in respect to time.


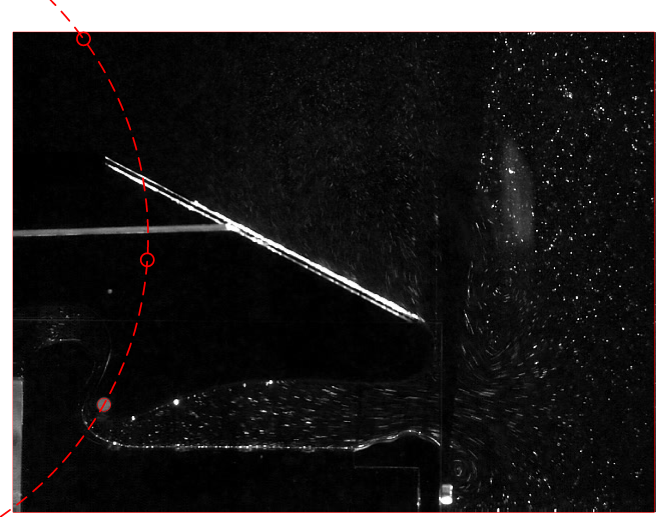

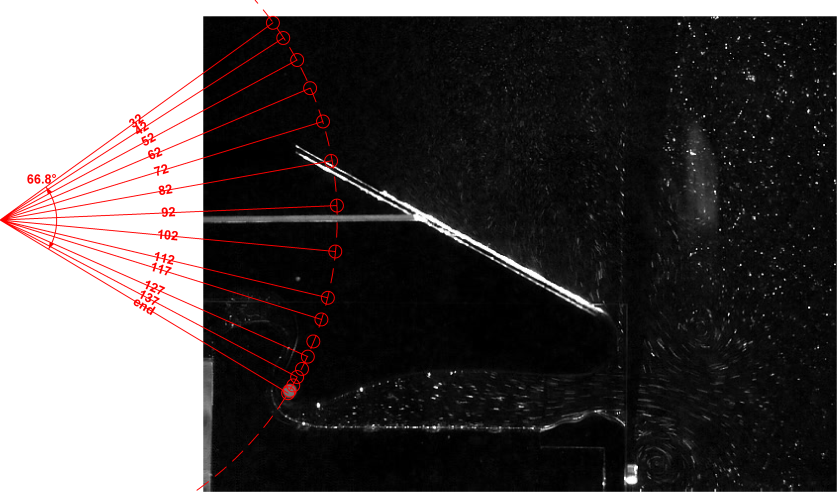


(a)

(b)

Figure 5. (a) Identification of the path (arc) of the white dot during closure. (b) identification of angle in relation to the closed position (*end*). The number corresponding to each line indicates the high-speed video frame used to obtain each angle measurement. Note that not all frames are shown, for clarity.

The angle/angular velocity data obtained are shown in Table A-1, or represented as a graph in Figure 6. Note that at the beginning of the high speed video the plunger is not visible, since it is outside the camera field of view. For this reason, extrapolation of the angular velocity was performed; extrapolated values are indicated with italics in Table 1. It has to be mentioned that the temporal resolution of the video capture was somewhat low for the plunger closure speed. This can be understood considering that:

- the maximum angular velocity was ~ 4 rad/s and the radius of rotation at the white marker was ~0.1 m, thus the maximum marker velocity was ~0.4m/s.

- the video resolution was 640 x 480 pixels for a frame size of 196 x 146 mm (W x H), i.e. the spatial resolution of the video frames was ~0.3 mm/pixel.

- within one frame the white marker maximum displacement is 0.4/300 = 1.33 mm which is ~ 4.5 pixels.

Thus in several frames, especially when the plunger moves at maximum speed, the white marker appeared blurred and consequently there is uncertainty in the closure angle and angular velocity.

The closure profile, applies for the 'enlarged scale' model used in the experiments. The 'enlarged scale' model and closure profile will be used as a validation case and for analyzing the main flow mechanisms during claw closure. However, by design, the 'enlarged scale' model was not expected to cavitate in the experiment performed and thus it is not expected to do so in simulation as well. For this reason, a 'real size' geometry is analyzed in the present work, scaled properly in respect to actual pistol shrimp claw (see Table 1). The closure profile for the 'real size' models is derived based on the closure angle of Table A-1, scaled at closure time of 0.3, 0.4 and 0.5 ms, instead of 0.5 s for the 'enlarged scale' model (see Figure 7), based on similarity.

Table A-1. Plunger angle and angular velocity, during closure

| Frame instance | Time | Angle | | Angular velocity |
| --- | --- | --- | --- | --- |
|  | (s) | (deg) | (rad) | (rad/s) |
| *0* | *0* | *73* | *1.274* | *0* |
| *16* | *0.053* | *71.1* | *1.241* | *-1.25* |
| 32 | 0.107 | 66.8 | 1.166 | -1.62 |
| 42 | 0.14 | 63.7 | 1.112 | -1.96 |
| 52 | 0.173 | 59.3 | 1.035 | -2.54 |
| 62 | 0.207 | 54 | 0.942 | -2.96 |
| 72 | 0.24 | 48 | 0.838 | -3.38 |
| 82 | 0.273 | 41.1 | 0.717 | -3.79 |
| 92 | 0.307 | 33.5 | 0.585 | -4.06 |
| 102 | 0.34 | 25.6 | 0.447 | -4.14 |
| 112 | 0.373 | 17.7 | 0.309 | -4.12 |
| 117 | 0.39 | 13.8 | 0.241 | -4.14 |
| 122 | 0.407 | 9.8 | 0.171 | -3.51 |
| 127 | 0.423 | 7.1 | 0.124 | -2.62 |
| 132 | 0.44 | 4.8 | 0.0838 | -2.04 |
| 137 | 0.457 | 3.2 | 0.056 | -1.68 |
| 142 | 0.473 | 1.6 | 0.028 | -1.41 |
| 147 | 0.49 | 0.5 | 0.009 | -1.05 |
| 149 | 0.497 | 0.2 | 0.003 | -0.87 |
| 150 | 0.5 | 0 | 0 | 0 |


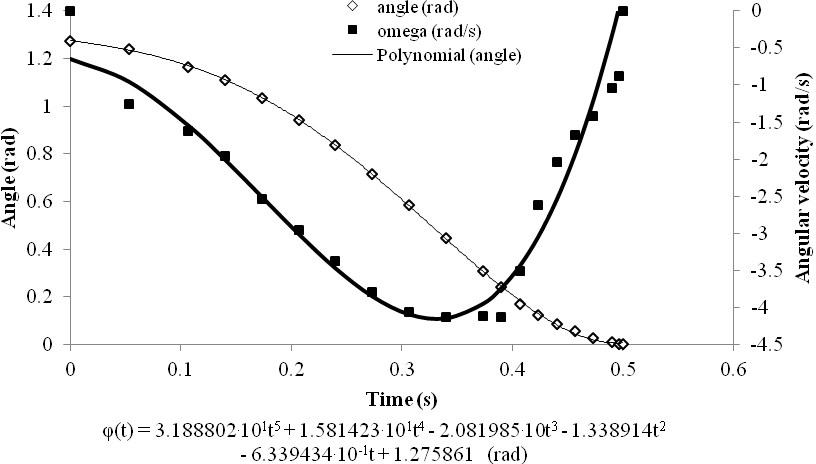


Figure 6. Plunger angle, *φ*, and closure speed (angular velocity, *ω*) for the enlarged configuration. Polynomial fit is provided for the angle.


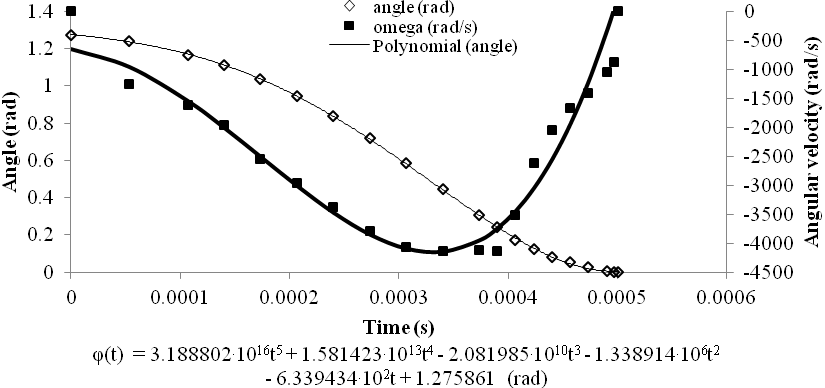


(a)

*
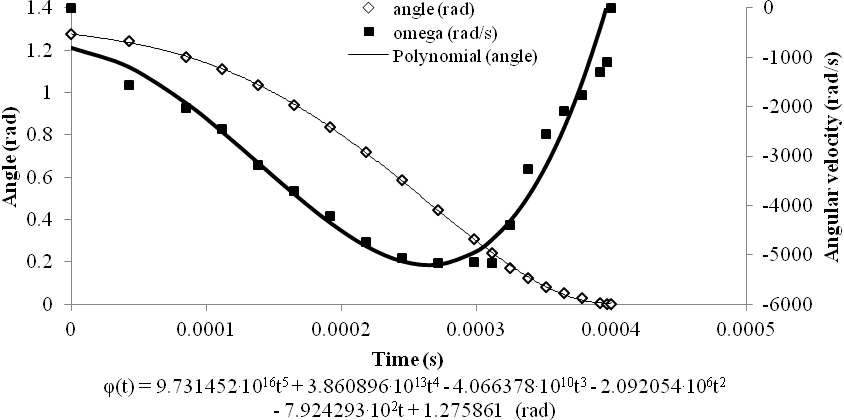
*

(b)


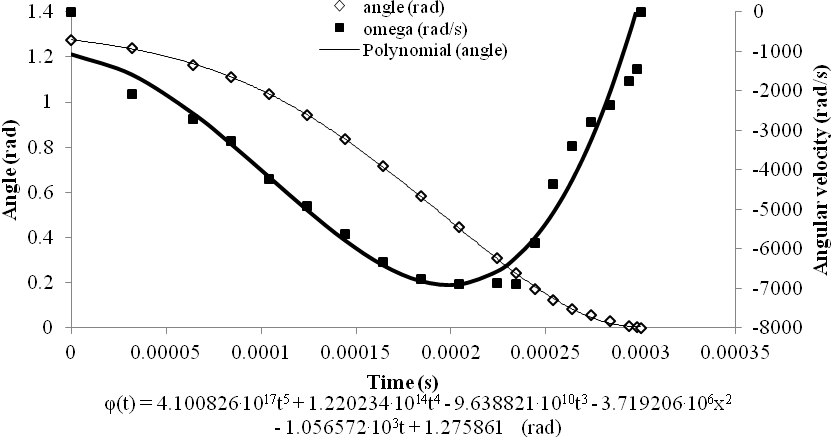


(c)

Figure 7. Plunger angle, *φ*, and closure speed (angular velocity, *ω*) for the 'real size' configuration; parametric studies for different closure speeds: (*a*) 0.5 ms, (*b*) 0.4 ms and (*c*) 0.3 ms. The closure speed is scaled based on the closure speed of the enlarged size experiment. Polynomial fit is provided for the angle for all cases.

**Supplementary material 4: Validation of the methodology for the 'enlarged model' case**

In this section the flow development in the enlarged plunger/socket configuration will be analyzed, approached with 3D simulations. Velocity profile at the neck of the claw is compared with experimental data from the experiment[^20^](#_ENREF_20). Figure 8 shows a comparison between the PIV measurements at the neck of the nozzle and the simulation results. The simulation predicts the correct trend of velocity profile, though it seems to be lagging behind the experimental data and to overpredict maximum velocity. We speculate that this is due to the uncertainty of the angle measurement from the video frames, since, as mentioned in *Supplementary material 3*, image blurring was observed at the maximum plunger speed. Another reason of the discrepancy between simulation and experimental data, is the inherent averaging inside the PIV interrogation window [^20^](#_ENREF_20), which may lead to under prediction of the actual velocity.


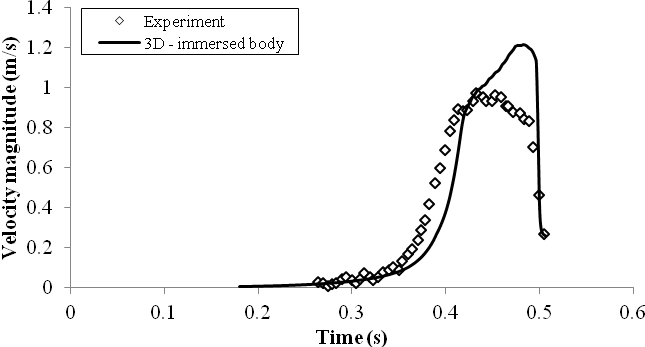


Figure 8. Comparison between the experiment and 3D numerical simulation.

Figure 9 shows the vortex ring motion over time, where it is visible that, apart from the motion toward the positive *y*-axis, there is a small velocity component towards the minus *z*-axis. The vortex ring size extent is estimated through the vortex core on the midplane of the geometry (see also Figure 11, showing *x*-vorticity at the midplane of the geometry). The vortex ring cross section is a Burgers vortex, which, soon after its formation, travels at approximately half the jet velocity. At the end of the simulation, the vortex translational velocity is ~0.34m/s. The predicted circulation of the vortex ring varies from ~0.0136 to 0.016m^2^/s, whereas in the experiment it was found to be 0.0128-0.014m^2^/s [^20^](#_ENREF_20). The predicted minor radius of the rigid body vortex core is found to be ~4 mm, as in the experiment [^20^](#_ENREF_20).

The flow field is presented in Figure 10. It is very similar to the one forming at the 'real size' simulations, due to Reynolds and Strouhal [^20^](#_ENREF_20) similarity. Vortical structures are indicated with the isosurface of the *q*-criterion (see ) for a value of 100 s^-2^. As the plunger starts to move, flow detachment occurs and two counter-rotating vortices are formed at the wake of the plunger, indicated with (*1*). As the plunger continues to move, these vortices become larger and start to twist, see (*2*) and (*3*). At 0.4s, the flow at the wake of the plunger is fully detached, exhibiting a set of complicated vortical structures. Note also that at the same instant, vortices start to form at the edges of the socket. As the plunger is further inserted in the socket, at 0.46s, the flow detaches at the sides of the plunger, as indicated by the twisted vortical structures denoted with (*5*). At the same instance, a high-speed jet is expelled towards the positive *y*-axis, from the opening between the plunger and the socket. A vortex ring forms around the jet, as indicated with (*6*); since the geometry is the extrusion of a 2-dimensional shape, the nozzle and the jet have a square cross-section, thus the vortex ring is also square shaped, at least in the beginning of its formation. After its formation, the vortex ring detaches from the socket/plunger opening and starts to move in the direction of the jet, see (*7*). The same mechanism has been observed in other experiments as well, see .


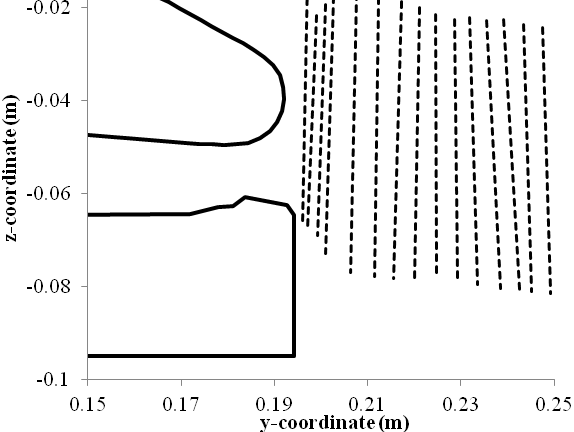


(a)

(b)

(c)

(d)

(e)

(a) t = 0.42s

(b) t = 0.5s

(c) t = 0.53s

(d) t = 0.57s

(e) t = 0.6

Figure 9. Indicative instances showing the vortex ring size (major radius) and position over time; the plunger/socket are shown at fully closed position. The maximum vortex propagation velocity at the *y*-axis is ~0.45m/s, around half of the maximum jet velocity in the inner part of the jet.


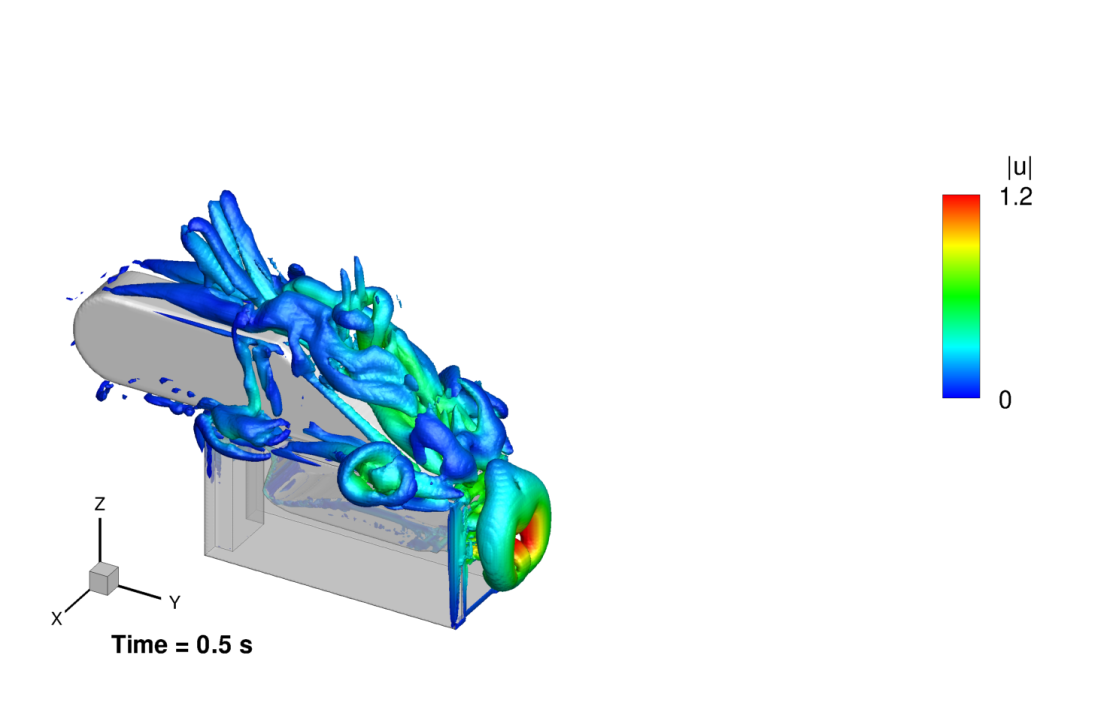

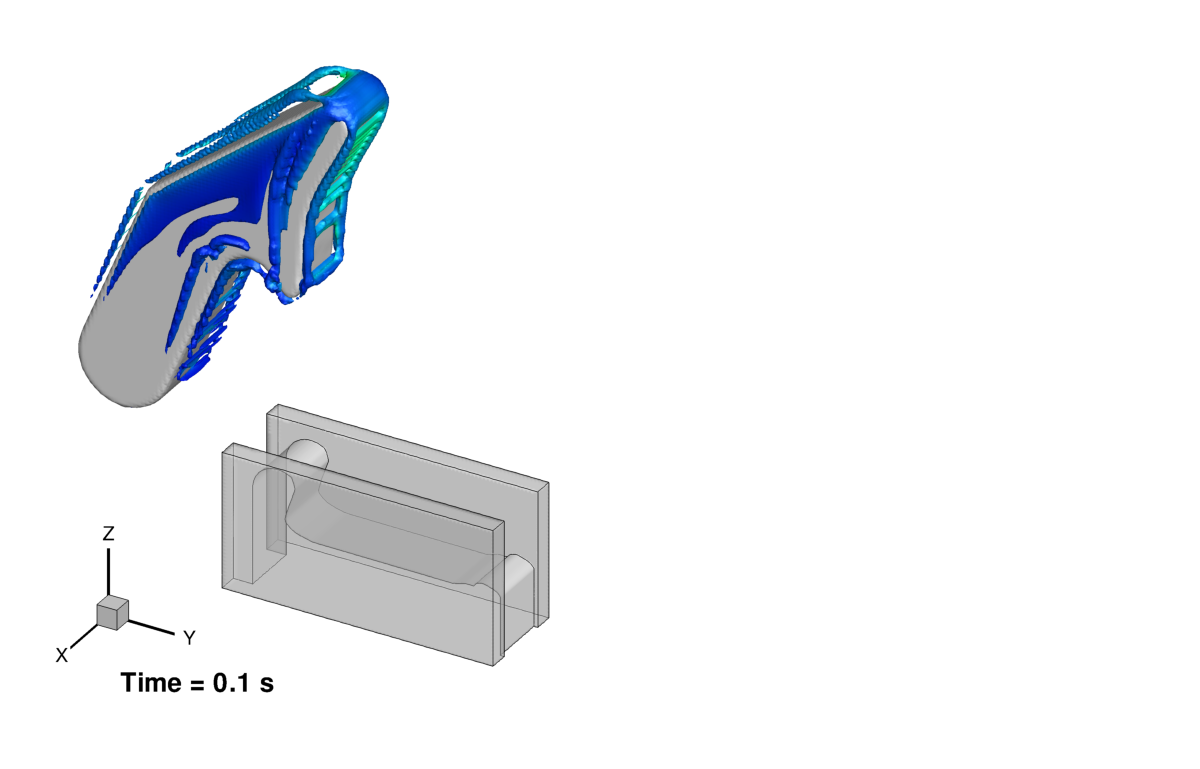

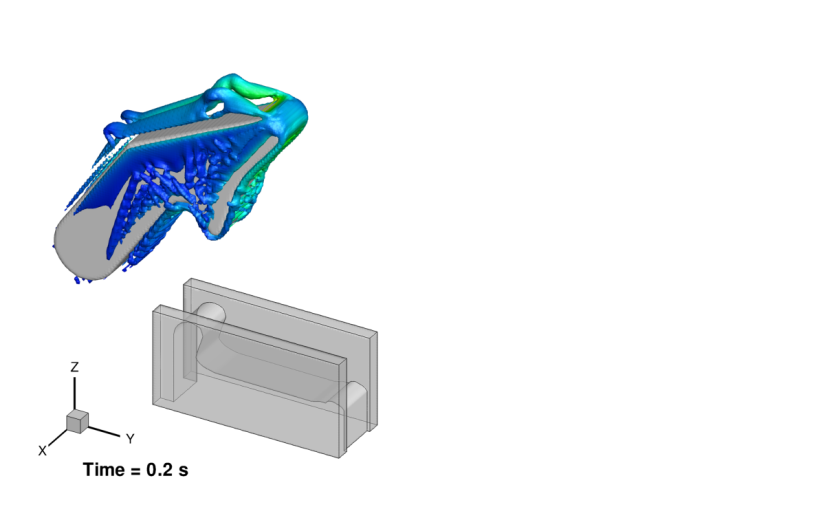

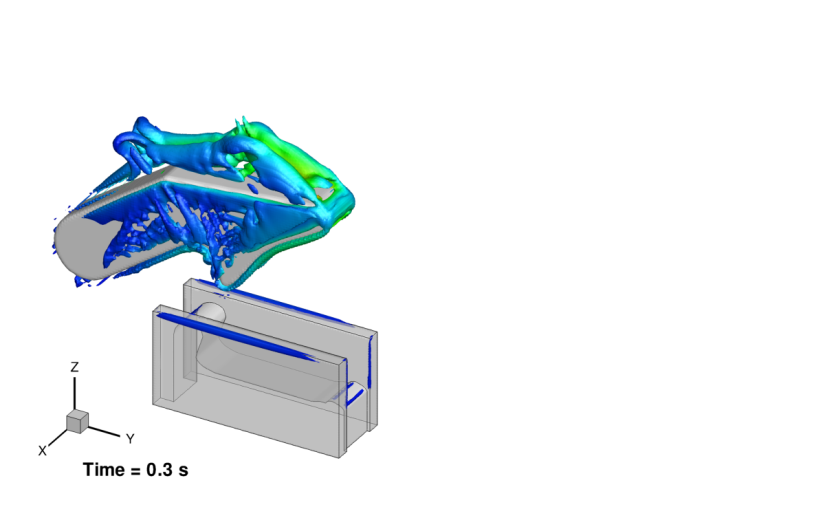


1

2

3

4


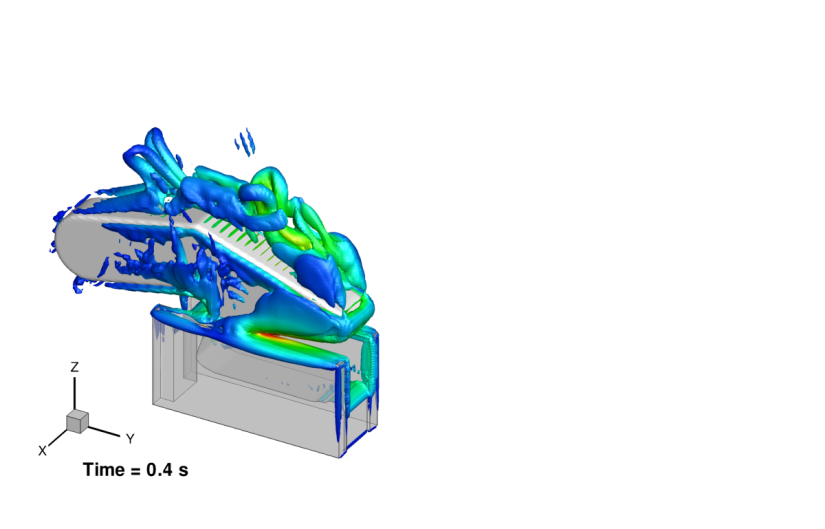

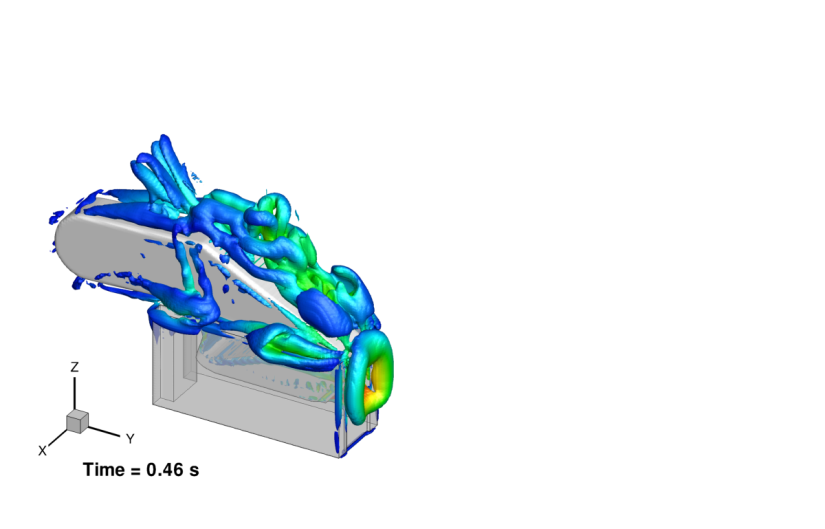

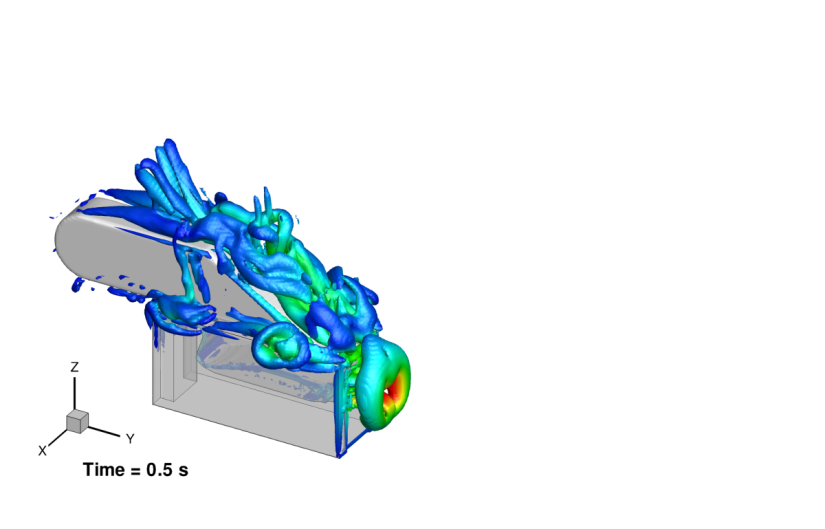


6

7

5

Figure 10. Indicative results of the vortical structures (indicated with the velocity gradient invariant *q* value of 100 s^-2^) coloured according to the velocity magnitude. Closure time 0.5 s.


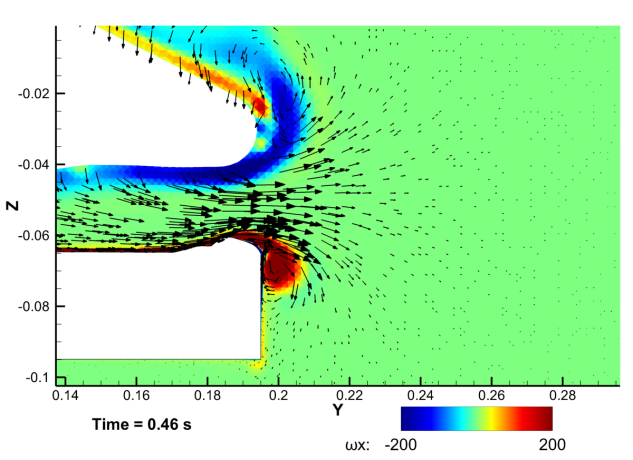

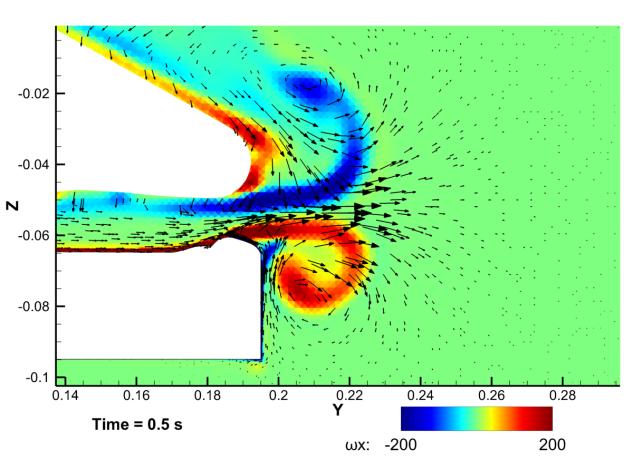

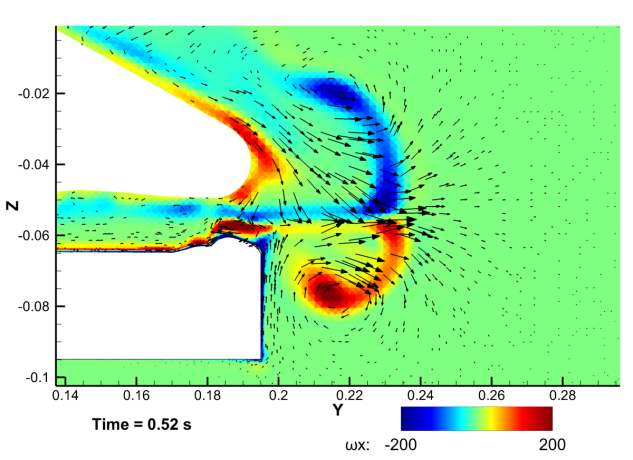

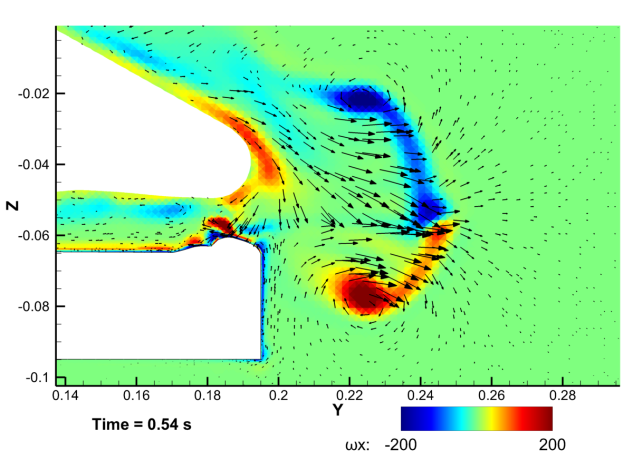


Figure 11. *x*-vorticity (*ω_x_*, 1/s) and vector plot along the midplane (*yz*-plane) of the geometry. The formation and propagation of the vortex ring is visible. Only one every forty vectors is shown for clarity.

**Supplementary material 5: Mesh convergence of the results**

In this section indicative results will be presented, demonstrating the mesh convergence of the simulations. The case under consideration is the 'enlarged geometry', as examined in the previous section (*Supplementary material 4*), since there are experimental data to compare against. Three levels of refinement were employed, each one having half the cell dimensions of the previous step. For each case the time step was adjusted accordingly, to maintain a convective Courant number of ~ 0.1, meaning that progressive spatial refinement leads to temporal refinement as well. An indicative table summarizing the resolution in different areas is provided below:

Table A-2. Plunger angle and angular velocity, during closure

| *Case* | **Coarse**  (2^.^*dx*) | **Medium**  (*dx*) | **Fine**  (*dx*/2) |
| --- | --- | --- | --- |
| *Base resolution (mm)* | 28 | 14 | 7 |
| *Plunger refinement (mm)* | 3.5 | 1.75 | 0.875 |
| *Socket refinement (mm)* | 1.4 | 0.7 | 0.35 |
| *Time step (μs)* | 100 | 50 | 25 |

The convergence study is assessed in terms of velocity profile, measured at the neck of the plunger/socket. Results for the different resolutions are shown in Figure 12. As shown, during the acceleration period, from 0.2-0.4s, the flow velocity is essentially identical for all cases. There is a minor variation for the peak velocity (max. velocity for coarse: 1.24m/s, medium: 1.21m/s, fine: 1.2m/s), however, the deviation between the successive refinement levels is less than 2%. The convergence order of the simulation, *p*, may be estimated as [^24^](#_ENREF_24):

$p=\ln\left(\frac{f_{coarse}-f_{medium}}{f_{medium}-f_{fine}}\right)/\ln\left(r\right)$ (11)

where *f* is a variable of interest (e.g. in the present case the peak velocity of the jet) and *r* is the refinement ratio (in our case *r* = 2). The convergence ratio for the levels of refinement used is ~1.6. Given the aforementioned results, it is argued that the results are mesh independent and the resolution of the mesh used (the 'medium' resolution, see Table A-2) is enough to describe the main physical mechanisms.


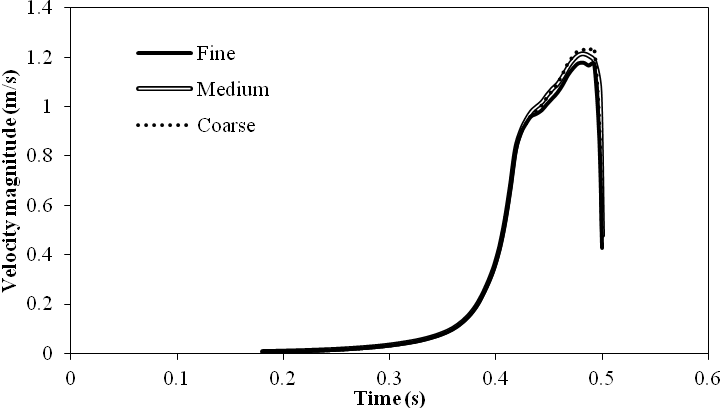


Figure 12. Comparison of the velocity magnitude variation at the neck of the plunger/socket nozzle over time, for the three refinement levels, discussed in

**Supplementary material 6: summary of the spatial/temporal resolution for the cases examined**

Table A-3. Summary of spatial/temporal resolution for all examined cases. (a), (b), (c) correspond to real size closure speed 0.5ms, 0.4ms and 0.3ms respectively.

|  | **Simulations** | | **Convergence study** | | |
| --- | --- | --- | --- | --- | --- |
|  | **Enlarged** | **Real size** | **Coarse** | **Medium** | **Fine** |
| *Spatial resolution* |  | |  | | |
| Base | 14mm | 0.2mm | 28mm | 14mm | 7mm |
| Plunger region | 1.75mm | 25μm | 3.50mm | 1.75mm | 0.87mm |
| Socket surface | 0.7mm | 10μm | 1.4mm | 0.7mm | 0.35mm |
| Refinement ratio (in relation to 'enlarged' case) | 1 | 1/70 | 2 | 1 | 0.5 |
| *Temporal resolution* |  | |  | | |
| Time step size | 50μs | (a): 50ns | 0.1ms | 50μs | 25μs |
|  |  | (b): 40ns |  |  |  |
|  |  | (c): 30ns |  |  |  |
| *Number of time steps* | 10000 | | 5000 | 10000 | 20000 |

**Supplementary material 7: flow reversal inside the simplified claw cavity**


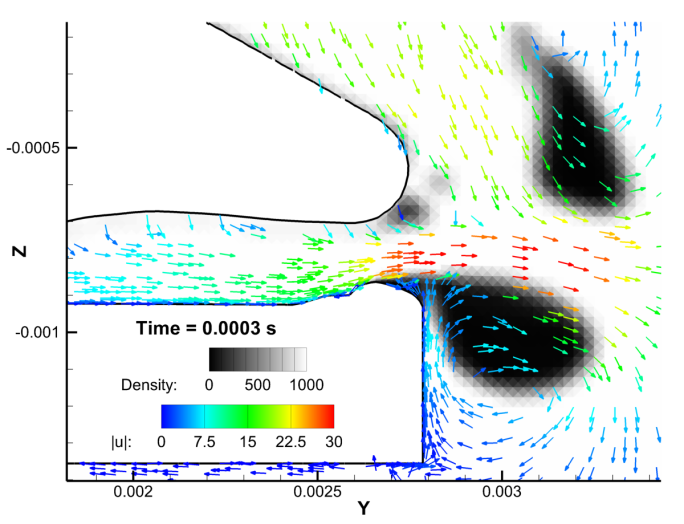

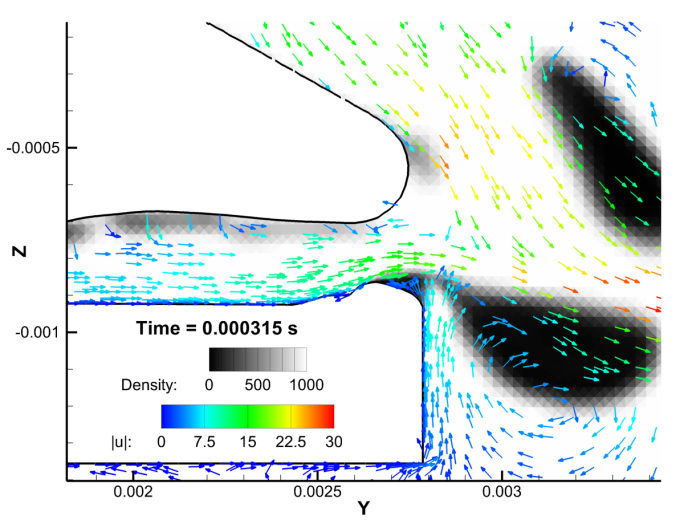

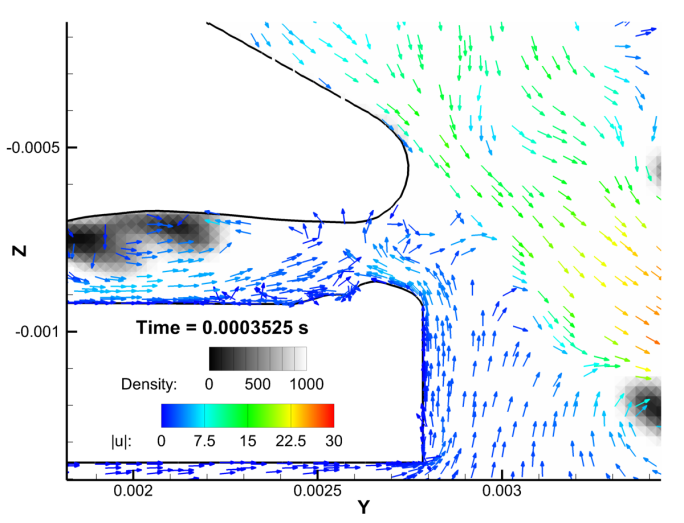

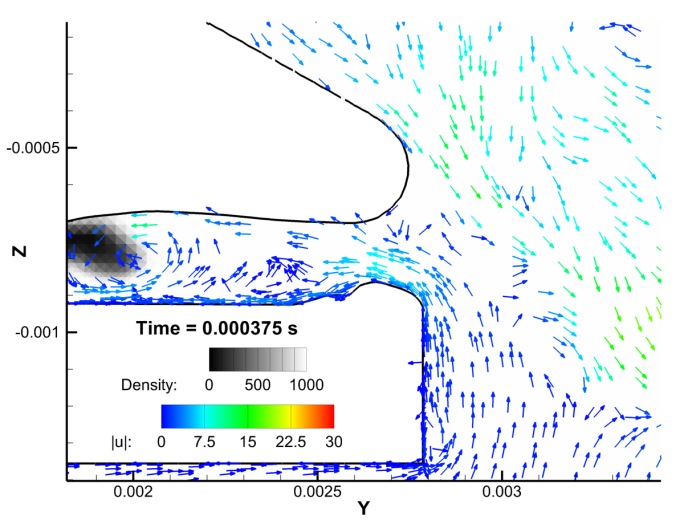


Figure 13. Flow reversal demonstrated for plunger closure at 0.3ms. Vectors are plotted at the midplane of the geometry. Note that all vectors have the same length, velocity magnitude is indicated as vector colour. The thick black line represents the plunger (immersed body). Density is represented as greyscale (black corresponds to vapour and white to liquid).

**Supplementary material 8: enlarged_size.avi**

Indicative results of the vortical structures (indicated with the velocity gradient invariant, *q*, value of 100 s^-2^) coloured according to the velocity magnitude. Closure time 0.5 s, max. plunger angular velocity 4 rad/s, max. plunger velocity at tip ~ 0.4 m/s. The video is also provided in yellow-blue palette.

**Supplementary material 9: real_size_0.5ms.avi**

Indicative instances of the 'real size' claw model closure; closure time 0.5 ms, max. plunger angular velocity 4000 rad/s, max. plunger velocity at tip~ 5.7 m/s. Vortices are shown, represented with the velocity gradient second invariant (value *q*=10^8^ s^-2^), coloured according to the velocity magnitude (semi-translucent isosurface). Cavitation is shown with the density isosurface for a value of 990 kg/m^3^ (i.e. vapour vol. fraction ~1% - black opaque isosurface). The video is also provided in yellow-blue palette.

**Supplementary material 10: real_size_0.4ms.avi**

Indicative instances of the 'real size' claw model closure; closure time 0.4 ms, max. plunger angular velocity 5200 rad/s, max. plunger velocity at tip~ 7.4 m/s. Vortices are shown, represented with the velocity gradient second invariant (value *q*=10^8^ s^-2^), coloured according to the velocity magnitude (semi-translucent isosurface). Cavitation is shown with the density isosurface for a value of 990 kg/m^3^ (i.e. vapour vol. fraction ~1% - black opaque isosurface). The video is also provided in yellow-blue palette.

**Supplementary material 11: real_size_0.3ms.avi**

Indicative instances of the 'real size' claw model closure; closure time 0.3 ms, max. plunger angular velocity 7000 rad/s, max. plunger velocity at tip ~ 9.8 m/s. Vortices are shown, represented with the velocity gradient second invariant (value *q*=10^8^ s^-2^), coloured according to the velocity magnitude (semi-translucent isosurface). Cavitation is shown with the density isosurface for a value of 990 kg/m^3^ (i.e. vapour vol. fraction ~1% - black opaque isosurface). The video is also provided in yellow-blue palette.

**Supplementary material 12: 3D CAD geometry of the claw/socket.**

3D CAD geometry of the plunger/socket, in PARASOLID v.18 binary format. Two files are provided, both in ***mm***:

- geometry_enlarged.x_b, is the enlarged geometry used in the experiments of Hess et al.[^20^](#_ENREF_20)

- geometry_realsize.x_b, is the real size geometry (i.e. the enlarged geometry, scaled down 70 times).

The files are checked to be compatible with the following CAD software: ANSA v.17.1, Solidworks 2016, Gambit 2.4.6, ANSYS 17.2 Design Modeller. The geometry should be also compatible with any other major CAD software, though this was not tested.

The files may be downloaded from:

https://grabcad.com/library/pistol-shrimp-simplified-claw-model-2

**Supplementary material 13: manuscript figures in colour-blind palette**

In this section, figures from the main text are provided in yellow/blue palette suitable, for individuals affected by protanopia/deuteranopia colour-blindness. These images are better suited for greyscale representation/printing.

Figure 2:


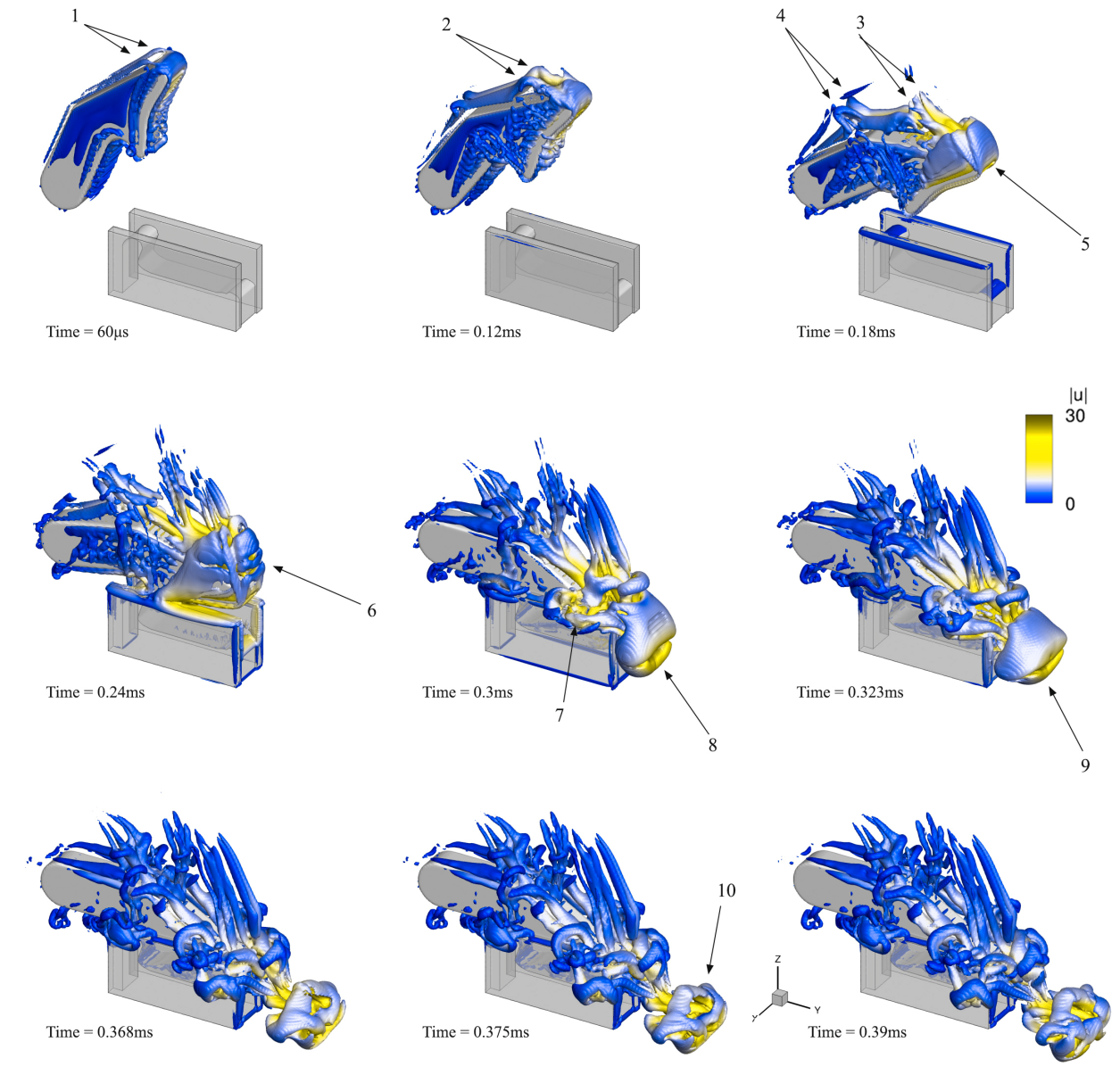


. Indicative instances of the 'real size' claw model closure; closure time 0.3 ms. Vortices are shown, represented with the velocity gradient second invariant (value *q*=10^8^ s^-2^), coloured according to the velocity magnitude.

Figure 3:


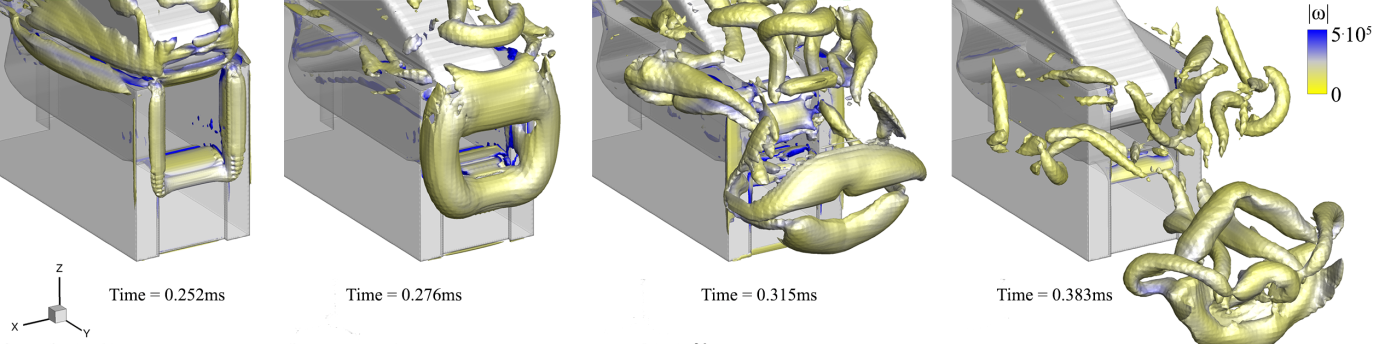


Indicative instances of the vortex ring formation, vortical structures indicated using a q-criterion value of 5.10^9^ s^-2^. The isosurface is colored according to local vorticity magnitude, providing an indication of the swirling angular velocity. Note that due to the square opening between plunger and socket, the vortex ring has initially a square shape as well.

Figure 4:


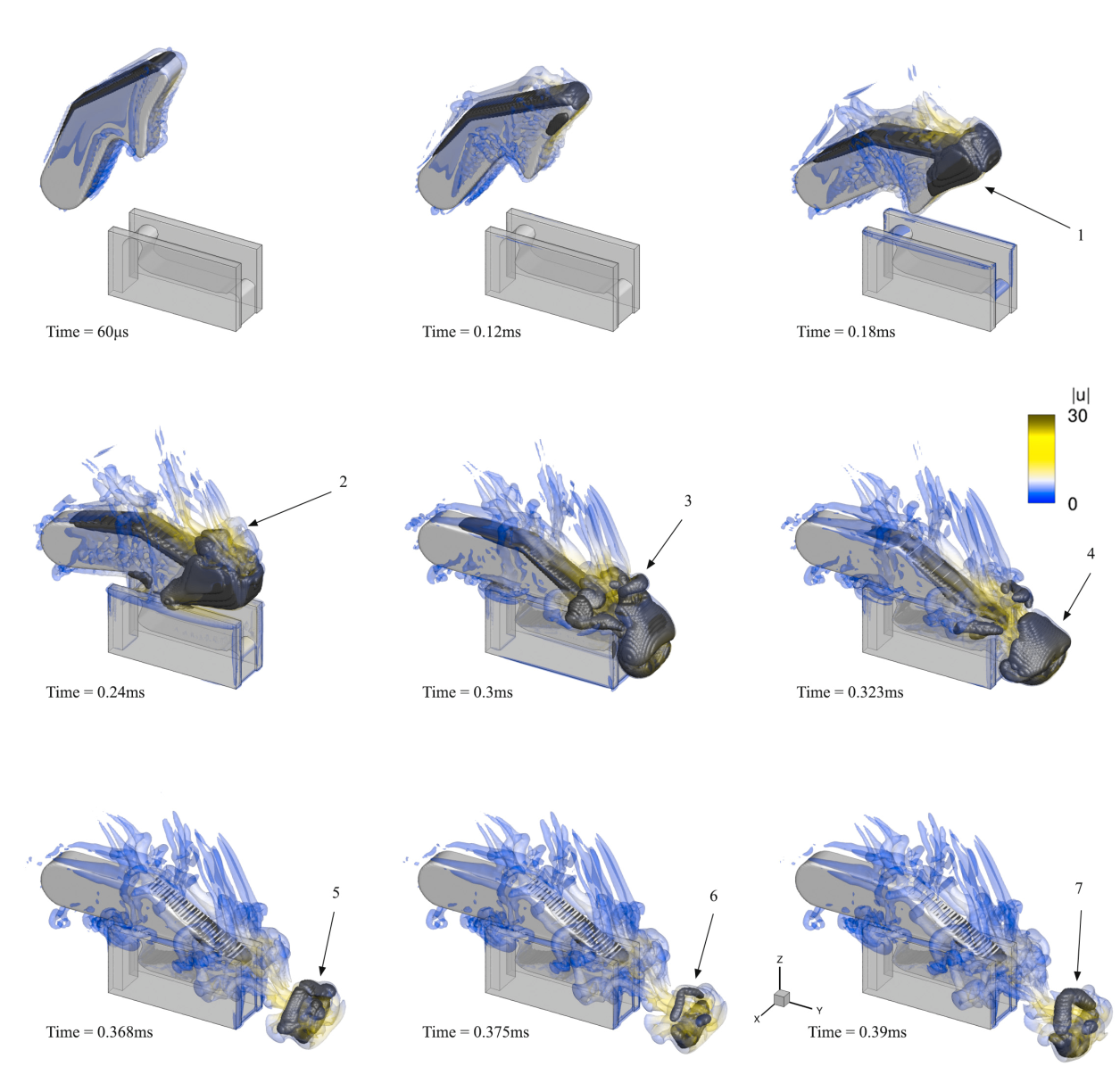


Indicative instances of the simplified claw model closure; closure time 0.3 ms. Vortices are shown, represented with the velocity gradient second invariant (value *q*=10^8^ s^-2^), coloured according to the velocity magnitude (semi-translucent isosurface). Cavitation is shown with a density isosurface for a value of 990 kg/m^3^ (i.e. vapour vol. fraction ~1% - black opaque isosurface).

Figure 5:


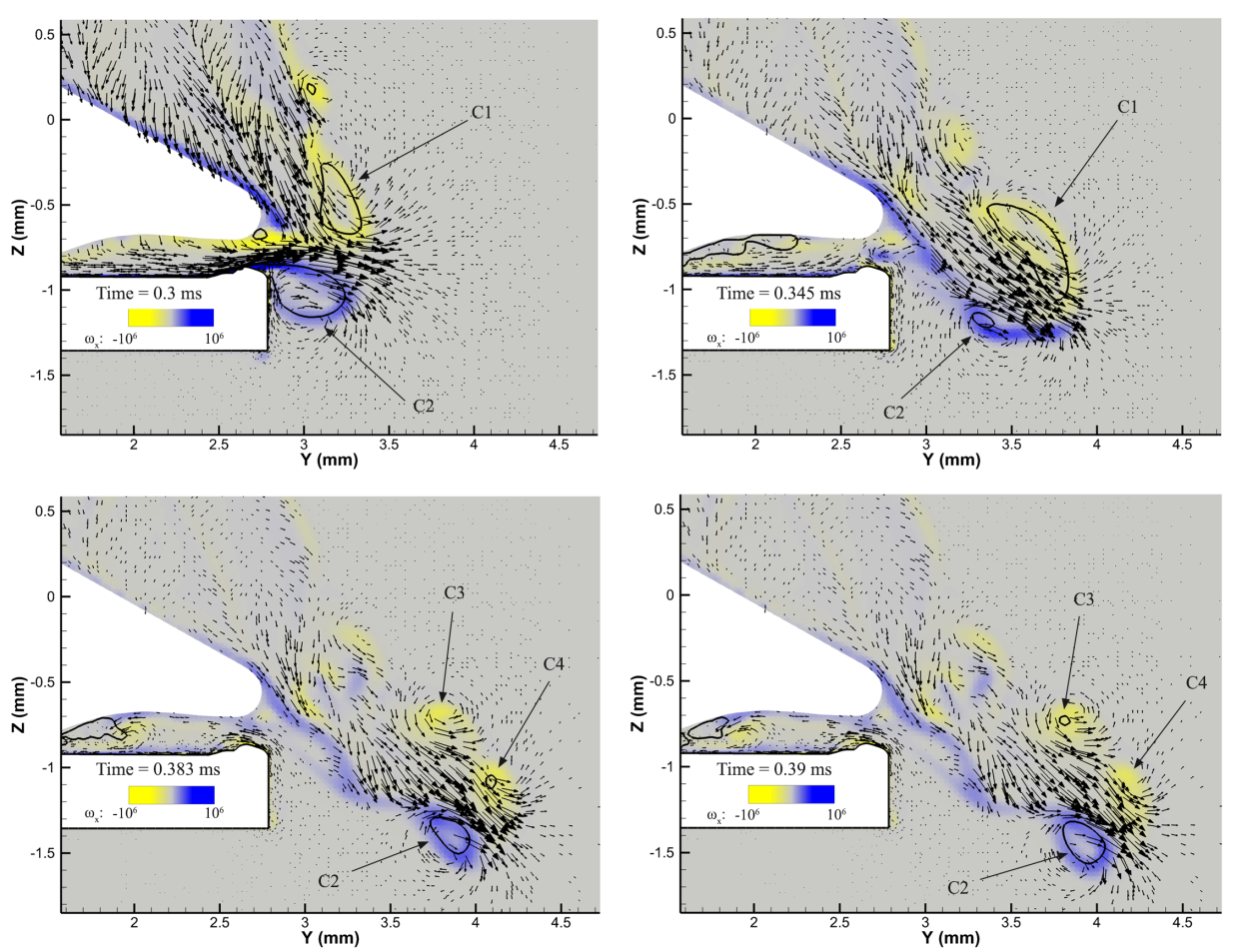


*x*-vorticity (*ω_x_*, 1/s) and velocity vectors represented at the midplane (*yz*-plane) of the geometry. The black thick line indicates a density isoline of 500 kg/m^3^ (i.e. vapour volume fraction of 50%).

Figure. 8:


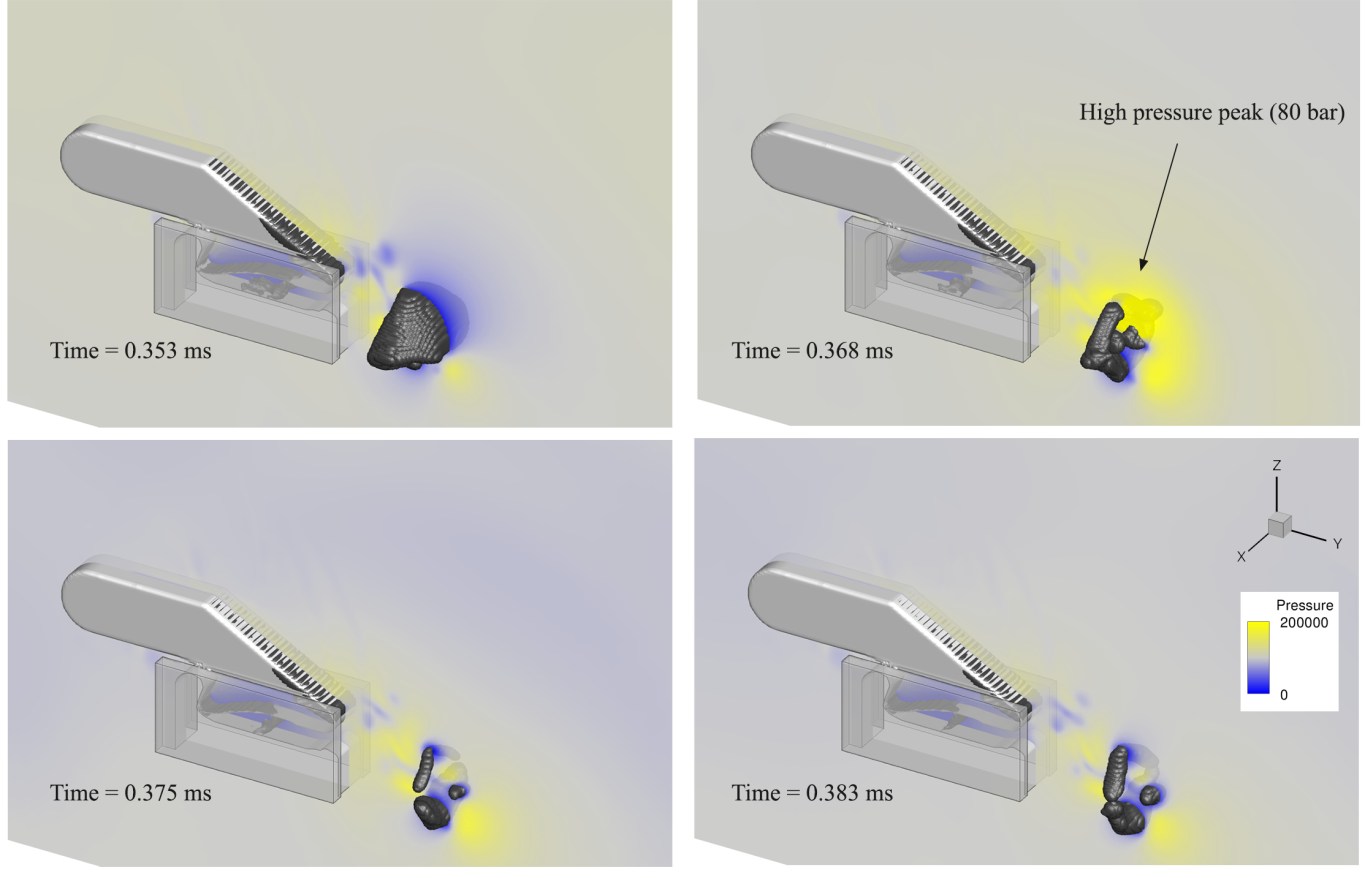


Pressure peak due to cavity collapse, plunger closure at 0.3ms. Pressure is shown at a midplane slice. The black isosurface is the 1% vapour fraction. Pressure, locally, may exceed 80bar.

**References**

1 Adams, N. A. & Schmidt, S. J. in *Bubble dynamics & Shock waves* (ed C. F. Delale) 235-256 (Springer-Verlag, 2013).

2 Sezal, I. H., Schmidt, S. J., Schnerr, G. H., Thalhamer, M. & Förster, M. Shock and wave dynamics in cavitating compressible liquid flows in injection nozzles. *Shock Waves* **19**, 49-58, doi:10.1007/s00193-008-0185-3 (2009).

3 Koukouvinis, P. & Gavaises, M. Simulation of throttle flow with two phase and single phase homogenous equilibrium model. *Journal of Physics: Conference Series* **656**, 012086, doi:10.1088/1742-6596/656/1/012086 (2015).

4 Koukouvinis, P., Naseri, H. & Gavaises, M. Performance of Turbulence and Cavitation Models in Prediction of Incipient and Developed Cavitation. *International Journal of Engine Research* (2016).

5 Zwart, P. J., Gerber, A. G. & Belamri, T. in *5th International Conference on Multiphase Flow* (Yokohama, Japan, 2004).

6 Schnerr, G. H. & Sauer, J. in *Fourth International Conference on Multiphase Flow* (New Orleans, USA, 2001).

7 Batchelor, G. K. *An Introduction to Fluid Dynamics*. (Cambridge University Press, 2000).

8 De Tullio, M. D., Cristallo, A., Balaras, E. & Verzicco, R. Direct numerical simulation of the pulsatile flow through an aortic bileaflet mechanical heart valve. *Journal of Fluid Mechanics* **622**, 259–290, doi:10.1017/S0022112008005156 (2009).

9 Peskin, C. S. Flow patterns around heart valves: A numerical method. *Journal of Computational Physics* **10**, 252-271 (1972).

10 Mittal, R. & Iaccarino, G. Immersed boundary methods. *Annual Review of Fluid Mechanics* **37**, 239-261, doi:10.1146/annurev.fluid.37.061903.175743 (2005).

11 Egerer, C., Hickel, S., Schmidt, S. & Adams, N. Large-eddy simulation of turbulent cavitating flow in a micro channel. *Physics of Fluids* **26**, 30, doi:10.1063/1.4891325 (2014).

12 Sinibaldi, E., Beux, F. & Salvetti, M. V. A numerical method for 3D barotropic flows in turbomachinery. *Flow Turbulence Combustion* **76**, 371-381, doi:10.1007/s10494-006-9025-7 (2006).

13 Pouffary, B. Numerical Modelling of Cavitation. (2006).

14 X-steam v2.6, http://xsteam.sourceforge.net/.

15 Ivings, M. J., Causon, D. M. & Toro, E. F. On Riemann solvers for compressible liquids. *International Numerical Methods for Fluids* **28**, 395-418, doi:10.1002/(SICI)1097-0363(19980915)28:3<395::AID-FLD718>3.0.CO;2-S (1998).

16 *ANSYS Inc., Fluent 16.2 Manual*. (2015).

17 Chen, Z. J. & Przekwas, A. J. A coupled pressure-based computational method for incompressible/compressible flows. *Journal of Computational Physics* **229**, 9150–9165, doi:10.1016/j.jcp.2010.08.029 (2010).

18 Mencinger, J. in *Finite Volume Method - Powerful Means of Engineering Design* (ed Radostina Petrova) (InTech, 2012).

19 Thompson, J. F., Soni, B. K. & Weatherill, N. P. *Handbook of Grid Generation*. 1st edn, 1136 (CRC Press, 1998).

20 Hess, D., Bruecker, C., Hegner, F., Balmert, A. & Bleckmann, H. Vortex Formation with a Snapping Shrimp Claw *PLOS ONE* **8**, 10 (2013).

21 Green, M. A., Rowley, C. W. & Haller, G. Detection of Lagrangian Coherent Structures in 3D Turbulence. *Journal of Fluid Mechanics* **572**, 111-120 (2007).

22 Haller, G. An objective definition of a vortex. *Journal of Fluid Mechanics* **525**, 1-26, doi:10.1017/S0022112004002526 (2005).

23 Belyakov, G. B. & Filippov, A. N. Cavitating Vortex Generation by a Submerged Jet. *Journal of Experimental and Theoretical Physics* **102**, 862–868, doi:10.1134/S1063776106050177 (2006).

24 Roache, P. J. Quantification of uncertainty in computational fluid dynamics. *Annual Review of Fluid Mechanics* **29**, 123-160, doi:10.1146/annurev.fluid.29.1.123 (1997).
